# Weighted Multiplex Networks

**DOI:** 10.1371/journal.pone.0097857

**Published:** 2014-06-06

**Authors:** Giulia Menichetti, Daniel Remondini, Pietro Panzarasa, Raúl J. Mondragón, Ginestra Bianconi

**Affiliations:** 1 Department of Physics and Astronomy and INFN Sez. Bologna, Bologna University, Bologna, Italy; 2 School of Business and Management, Queen Mary University of London, London, United Kingdom; 3 School of Electronic Engineering and Computer Science, Queen Mary University of London, London, United Kingdom; 4 School of Mathematical Sciences, Queen Mary University of London, London, United Kingdom; University of Zaragoza, Spain

## Abstract

One of the most important challenges in network science is to quantify the information encoded in complex network structures. Disentangling randomness from organizational principles is even more demanding when networks have a multiplex nature. Multiplex networks are multilayer systems of 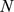 nodes that can be linked in multiple interacting and co-evolving layers. In these networks, relevant information might not be captured if the single layers were analyzed separately. Here we demonstrate that such partial analysis of layers fails to capture significant correlations between weights and topology of complex multiplex networks. To this end, we study two weighted multiplex co-authorship and citation networks involving the authors included in the American Physical Society. We show that in these networks weights are strongly correlated with multiplex structure, and provide empirical evidence in favor of the advantage of studying weighted measures of multiplex networks, such as multistrength and the inverse multiparticipation ratio. Finally, we introduce a theoretical framework based on the entropy of multiplex ensembles to quantify the information stored in multiplex networks that would remain undetected if the single layers were analyzed in isolation.

## Introduction

Network theory investigates the global topology and structural patterns of the interactions among the constituent elements of a number of complex systems including social groups, infrastructure and technological systems, the brain and biological networks [1]–[4]. Over the last fifteen years, a large body of literature has attempted to disentangle noise and stochasticity from non-random patterns and mechanisms, in an attempt to gain a better understanding of how these systems function and evolve. More recently, further advances in the study of complex systems have been spurred by the upsurge of interest in multiplex networks in which pairs of interacting elements are represented as nodes connected through multiple types of links, at multiple points in time, or at multiple scales of resolution [5]. More specifically, a multiplex network is a set of 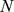 nodes interacting in 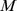 layers, each reflecting a distinct type (or time or resolution) of interaction linking the same pair of nodes. Examples of multiplex networks include: social networks, where the same individuals can be connected through different types of social ties originating from friendship, collaboration, or family relationships [6]; air transportation networks, where different airports can be connected through flights of different companies [7]; and the brain, where different regions can be seen as connected by the functional and structural neural networks [8].

Most of the studies so far conducted on multiplex networks have been concerned with the empirical analysis of a wide range of systems [6], [7], [9], [10], the modeling of their underlying structures [11]–[13], and the description of new critical phenomena and processes occurring on them [14]–[17]. Despite the growing interest in multiplex networks, a fundamental question still remains largely unanswered: What is the advantage of a full-fledged analysis of complex systems that takes all their interacting layers into account, over more traditional studies that represent such systems as single networks with only one layer? To answer this question, one should demonstrate that novel and relevant information can be uncovered only by taking the multiplex nature of complex systems directly into account, and would instead remain undetected if individual layers were analyzed in isolation. In this paper, an attempt is made to offer a possible solution to this problem within the context of weighted multiplex networks.

Just as with single networks, links between nodes may have a different weight, reflecting their intensity, capacity, duration, intimacy or exchange of services [18]. The role played by the weights in the functioning of many networks, and especially the relative benefits of weak and strong ties in social networks, have been the subject of a longstanding debate [18]–[20]. Moreover, it has been shown that, in single networks, the weights can be distributed in a heterogeneous way, as a result of the non-trivial effects that the structural properties of the networks have on them [21]–[24]. In particular, correlations between weights and structural properties of single networks can be uncovered by the analysis of strength-degree correlations [21] and by the distribution of the weights of the links incident upon the same node [23]. To characterize weighted networks, it is common practice to measure the following quantities: i) the average strength of nodes of degree 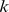, i.e. 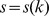, describing how weights are distributed in the network; and ii) the average inverse participation ratio of the weights of the links incident upon nodes of degree 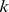, i.e. 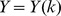, describing how weights are distributed across the links incident upon nodes of degree 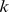. Here we show that these two quantities do not capture the full breadth of the information encoded in multiplex networks. Indeed, a full-fledged analysis of the properties of multiplex networks is needed that takes the multiple interacting and co-evolving layers simultaneously into account.

**Figure 1:**
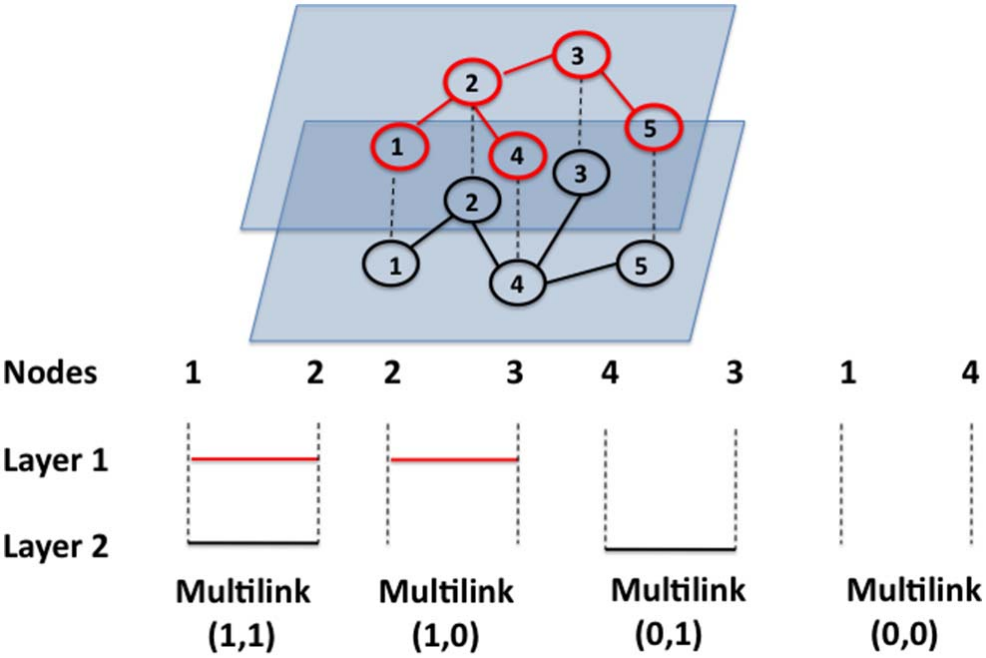
Example of all possible multilinks in a multiplex network with 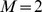 layers and 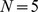 nodes. Nodes 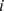 and 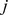 are linked by one multilink 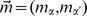.

For a multiplex network, a *multilink* 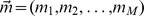 between nodes 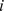 and 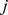 indicates the set of all links connecting these nodes in the different layers [25]. In particular, if 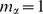, there is a link between nodes 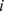 and 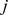 in layer 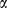, whereas if 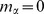 nodes 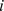 and 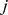 are not connected in layer 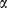. Multilink 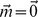 between two nodes refers to the case in which no link exists between the two nodes in all layers of the multiplex network. Thus, multilinks indicate the most straightforward type of correlation between layers, and provide a simple generalization of the notion of overlap. In fact, if nodes 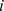 and 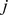 are connected by a multilink 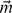, with 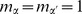, it follows that there is an overlap of links between 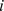 and 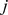 in layers 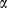 and 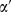. Figure 1 shows a multiplex network with 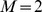 layers and 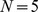 nodes with different types of multilinks.

Here we will define two new measures, *multistrength* and the *inverse multiparticipation ratio*, which are, respectively, the sum of the weights of a certain type of multilink incident upon a single node and a way for characterizing the heterogeneity of the weights of multilink 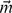 incident upon a single node. To provide empirical evidence that weighted properties of multilinks are fundamental for properly assessing weighted multiplex networks, we focus on the networks of the authors of papers published in the journals of the American Physical Society (APS), and analyze the scientific collaboration network and the citation network connecting the same authors. These networks are intrinsically weighted since any two scientists can co-author more than one paper and can cite each other’s work several times. A large number of studies have analyzed similar bibliometric datasets drawing upon network theory [26]–[30]. Unlike these studies, here we investigate the APS bibliometric dataset using the framework of multiplex networks that allows us to explore novel properties of the collaboration and citation networks. In particular, we show that multistrength and the inverse multiparticipation ratio enable new relevant information to be extracted from the APS dataset and that this information extends beyond what is encoded in the strength and inverse participation ratio of single layers. Finally, based on the entropy of multiplex ensembles, we propose an indicator 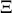 to evaluate the additional amount of information that can be extracted from the weighted properties of multilinks in multiplex networks over the information encoded in the properties of their individual layers analyzed separately.

## Weighted Multiplex Networks

### 2.1 Definition

A weighted multiplex network is a set of 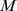 weighted networks 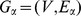, with 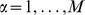. The set of nodes 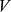 is the same for each layer and has cardinality 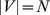, whereas the set of links 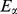 depends on the layer 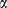. A multiplex network is represented formally as 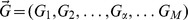. Each network 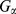 is fully described by the adjacency matrix 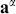 with elements 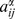, where 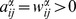 if there is a link with weight 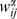 between nodes 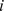 and 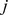 in layer 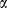, and 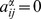 otherwise. From now on, in order to simplify the formalization of weighted multiplex networks, we will assume that the weight of the link between any pair of nodes 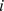 and 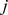, 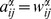, can only take integer values. This does not represent a major limitation because in a large number of weighted multiplex networks the weights of the links can be seen as multiples of a minimal weight.

### 2.2 Structural Properties of Individual Layers

We indicate the degree of node 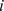 in layer 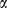 with 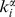, defined as 
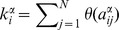
, where function 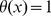 if 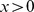, and 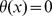 otherwise. In complex weighted networks, weights can be distributed across links more or less heterogeneously. A way to evaluate this heterogeneity is to introduce local properties such as the *strength* 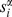 [21] and the *inverse participation ratio* 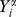 of node 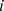 in layer 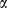 [22], [23]:
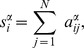


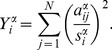
(1)As with single networks, in any given layer 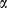, the strength 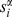 of a node indicates the sum of the weights of the links incident upon node 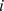 in layer 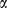, whereas the inverse participation ratio 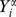 indicates how unevenly the weights of the links of node 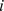 are distributed in layer 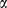. The inverse of 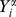 characterizes the effective number of links of node 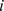 in layer 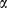. Indeed, 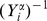 is greater than one and smaller than the degree of node 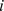 in layer 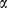, i.e., 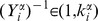. Moreover, if the weights of the links of node 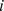 are distributed uniformly, i.e., 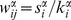, we have 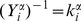. Conversely, if the weight of one link is much larger than the other weights, i.e., 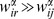 for every 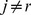, then 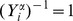.

In network theory, it is common practice to evaluate the conditional means of the strength and of the inverse participation ratio of the weights of links against the degree of nodes [21]–[23]. In a multiplex network, we will then consider the quantities 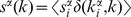 and 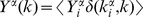, where the average is calculated over all nodes with degree 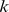 in layer 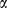, and 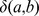 indicates the Kronecker delta. As in single networks [21], 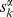 is expected to scale as
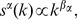
(2)with 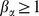. We can distinguish between two scenarios. In the first one, the average strength of nodes with degree 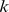 increases linearly with 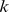, i.e., 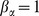. This indicates that, on average, the weights of the links incident upon the hubs do not differ from the weights of the links of less connected nodes. In the second scenario, the strength of the nodes with degree 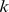 increases super-linearly with 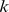, i.e., 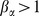, thus indicating that, on average, the weights of the links incident upon the hubs are larger than the weights of the links of less connected nodes. In a multiplex network, it may be the case that weights are distributed in different ways across the layers. For instance, some layers may be characterized by a super-linear growth of 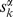, while other layers may show a linear dependence. Finally, the inverse participation ratio can be used in order to characterize the heterogeneity of the weights of the links incident upon nodes with a certain degree. In particular, it has been observed that, in many single weighted networks, the inverse participation ratio scales as an inverse power-law function of the degree of nodes. In a multiplex network, this would imply
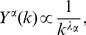
(3)where exponent 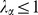 is layer-dependent.

### 2.3 Multilink, Multistrength, and Inverse Multiparticipation Ratio

A number of multiplex networks are characterized by a significant overlap of links across the different layers [6], [7]. In order to generalize the notion of overlap to weighted multiplex networks, in what follows we will draw on the concept of multilink [25]. Let us consider the vector 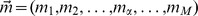, in which every element 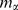 can take only two values 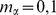. We define a *multilink* 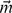 as the set of links connecting a given pair of nodes in the different layers of a multiplex network, and connecting them in the generic layer 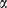 only if 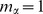. In particular, any two nodes 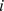 and 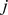 are always linked by a single multilink of type 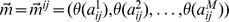, where 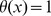 if 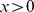, and 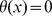 otherwise. The multilink 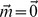 between two nodes represents the situation in which in all the layers of the multiplex network the two nodes are not directly linked.

We can now introduce the multiadjacency matrices 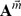 with elements 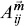 equal to 1 if there is a multilink 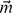 between node 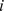 and node 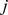 and zero otherwise. In terms of the weighted adjacency matrices 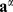 of the multiplex network, the elements 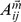 of the multiadjacency matrix 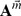 are given by
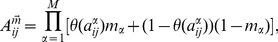
(4)where 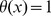 if 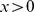, otherwise 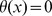. Even though there are 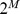 multiadjacency matrices, only 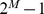 of them are independent because the normalization condition, 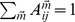, must be satisfied for any pair of nodes 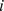 and 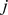. Based on multi-adjacency matrices, we can define the *multidegree* 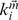 of node 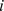 as
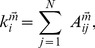
(5)which indicates how many multilinks 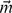 are incident upon node 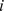.

To study weighted multiplex networks, we now introduce two new measures. For layer 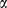 associated to multilinks 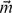, such that 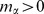, we define the multistrength 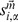 and the inverse multiparticipation ratio 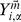 of node 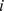, respectively, as
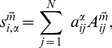
(6)

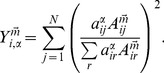
(7)


Since multistrength 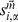 can be non-zero only if 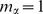, for each layer 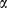 the number of non-trivial multistrengths is 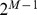, and therefore the number of multistrengths that can be defined in a multiplex network of 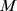 layers is 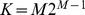. Similarly, the number of inverse multiparticipation ratios 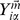 is given by 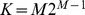. The average multistrength of nodes with a given multidegree, i.e., 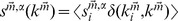, and the average inverse multiparticipation ratio of nodes with a given multidegree, i.e., 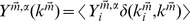, are expected to scale as
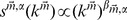


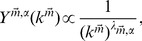
(8)with exponents 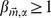 and 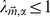. The use of multilinks 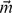 to describe multiplex properties is numerically feasible if the number of layers is smaller than the number of nodes, i.e., 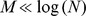. If this condition is not satisfied, then the following quantities can be measured: the *overlap multiplicity*, 
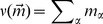
, which indicates that multilink 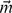 connects two nodes through 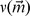 links; 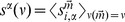; and 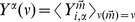, where 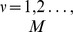.

### Empirical Evidence of Weighted Properties of Multilinks

In this section, we will draw on the measures introduced above and provide empirical evidence that, in weighted multiplex networks, weights can be correlated with the multiplex structure in a non-trivial way. To this end, we analyze the bibliographic dataset that includes all articles published in the APS journals (i.e., Physical Review Letters, Physical Review, and Reviews of Modern Physics) from 1893 to 2009. Of these articles, the dataset includes their citations as well as the authors. Here, we restrict our study only to articles published either in Physical Review Letters (PRL) or in Physical Review E (PRE) and written by ten or fewer authors, 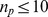. We constructed multiplex networks in which the nodes are the authors and links between them have a two-fold nature: scientific collaborations with weights defined as in [28] (see Text S1), and citations with weights indicating how many times author 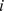 cited author 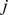.

In particular, we created the following two duplex networks (i.e., multiplex networks with 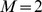):

1. **CoCo-PRL/PRE:** *collaborations among PRL and PRE authors.* The nodes of this multiplex network are the authors with articles published both in PRL and PRE (i.e., 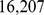 authors). These nodes are connected in layer 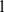 through weighted undirected links indicating the strength of their collaboration in PRL (i.e., co-authorship of PRL articles). The same nodes are connected in layer 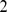 through weighted undirected links indicating the strength of their collaboration in PRE (i.e., co-authorship of PRE articles).
2. **CoCi-PRE:** *collaborations among PRE authors and citations to PRE articles.* The nodes of this multiplex network are the authors of articles published in PRE (i.e., 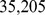 authors). These nodes are connected in layer 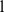 through weighted undirected links indicating the strength of their collaboration in PRE (i.e., co-authorship of PRE articles). The same nodes are connected in layer 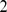 through weighted directed links indicating how many times an author (with articles in PRE) cited another author’s work, where citations are limited to those made to PRE articles.

Both these multiplex networks show a significant overlap of links and a significant correlation between degrees of nodes as captured by the Pearson correlation coefficient 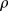 (see Text S1). This finding supports the hypothesis that the two layers in each of the multiplex networks are correlated. That is, the existence of a link between two authors in one layer is correlated with the existence of a link between the same authors in the other layer. Moreover, the multidegrees of the multiplex networks are broadly distributed, and the hubs in the scientific collaboration network tend to be also the hubs in the citation network (see Text S1).

**Figure 2:**
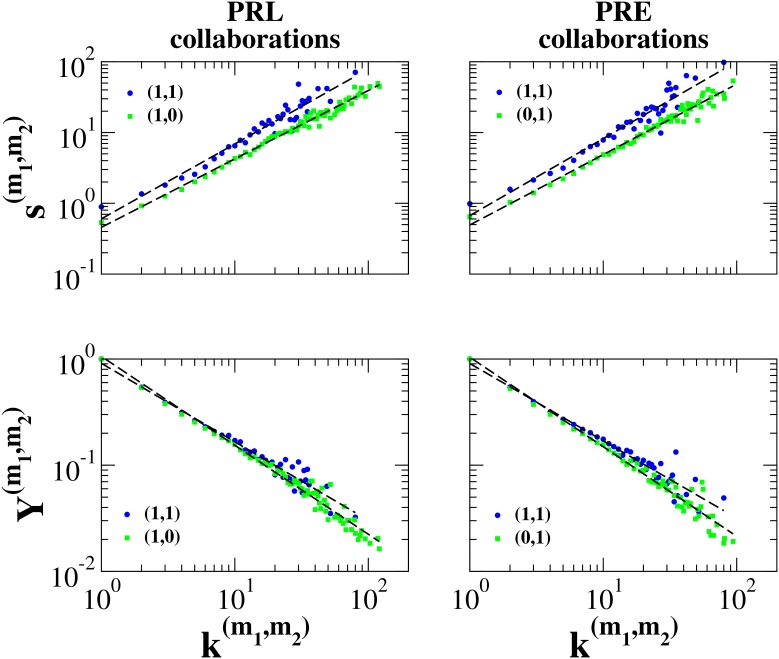
Average multistrength and average inverse multiparticipation ratio versus multidegree in the CoCo-PRE/PRL multiplex network. The average multistrengths and the average inverse multiparticipation ratios are fitted by a power-law distribution of the type described in Eq. (8) (fitted distributions are here indicated by black dashed lines). Statistical tests for the collaboration network of PRL suggest that the exponents 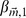 defined in Eq. (8) are the same, while exponents 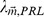 are significantly different. Similar results can be obtained for the exponents in the PRE collaboration layer. Nevertheless, multistrengths 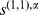 are always larger than multistrengths 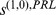 and 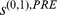, when multistrengths are calculated over the same number of multilinks, i.e., 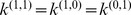 (see Text S1 for the statistical test on this hypothesis).

In the case of the CoCo–PRL/PRE network, multilinks 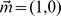, 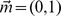 and 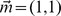 refer to collaborations only in PRL, only in PRE, and in both PRL and PRE, respectively. Moreover, to distinguish between the weights used when evaluating multistrength, we have 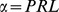 or 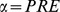. Results indicate that multistrength and the inverse multiparticipation ratio behave according to Eq. (8) (see Fig. 2). The difference between exponents 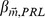 for 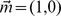 and 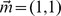 is not statistically significant. Nevertheless, there is a statistically significant difference between the average weights of multilinks 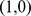 and 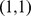 in the PRL layer. As to the inverse multiparticipation ratio, there is a significant variation in the exponents, 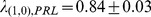 and 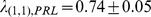 (see Fig. 2, bottom left panel). This suggests that the weights of the collaborative links between co-authors of both PRL and PRE articles are distributed more heterogeneously than the weights of collaborative links between co-authors of articles published only in PRL (see Text S1 for details on the statistical tests). Similar results were found for multistrengths evaluated in the PRE layer (see Fig. 2, right panels).

These findings clearly indicate that the partial analysis of individual layers would fail to uncover the fact that the average weight of the link between authors that collaborated both on PRL and PRE articles is significantly larger than the average weight of the link between authors that collaborated only on articles published in one journal. Moreover, the difference in functional behavior of the multipartition ratio across layers could not be captured if layers were analyzed separately.

**Figure 3:**
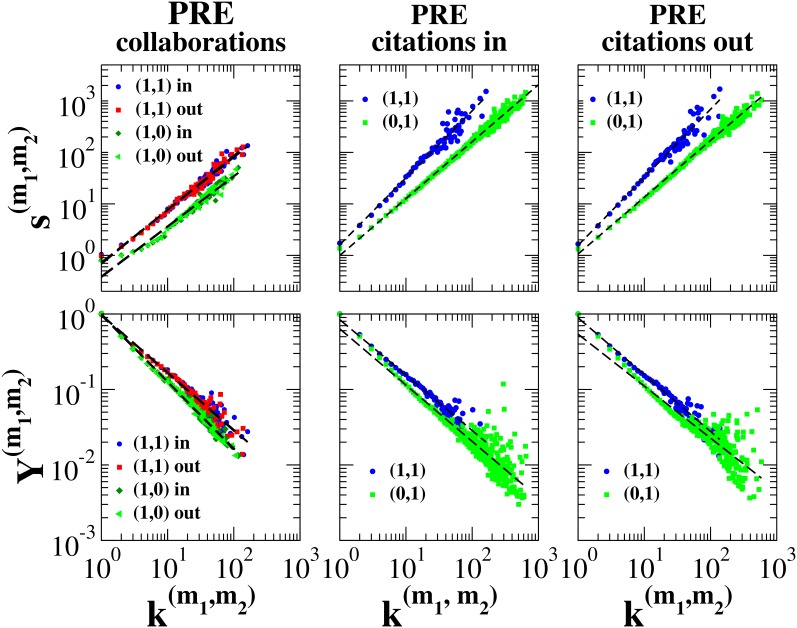
Properties of multilinks in the weighted CoCi-PRE multiplex network. In the case of the collaboration network, the distributions of multistrengths versus multidegrees always have the same exponent, but the average weight of multilinks 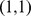 is larger than the average weight of multilinks 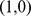. Moreover, the exponents 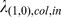, 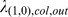 are larger than exponents 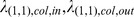. In the case of the citation layer, both the incoming multistrengths and the outgoing multistrengths have a functional behavior that varies depending on the type of multilink. Conversely, the average inverse multiparticipation ratio in the citation layer does not show any significant change of behavior when compared across different multilinks.

In the case of the CoCi-PRE network, there are even more significant differences between the properties of the multilinks than in the previous network. In the CoCi-PRE network the functional behavior of multistrength also depends on the type of multilink. Figure 3 shows the average multistrength in the CoCi-PRE network. To distinguish between the weights used to measure multistrength, we have layer 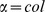, which refers to the collaboration network constructed on PRE articles, and layer 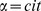, which refers to the citation network between PRE articles, where a distinction is also made between incoming (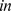) and outgoing (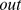) links. First, in the scientific collaboration network, exponents 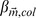 are not statistically different, but the average weight of multilink 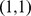 is larger than the average weight of multilinks 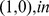 and 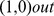. Moreover, exponents 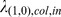 and 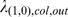 are larger than exponents 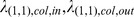, indicating that the weights of authors’ collaborative links with other cited/citing authors are distributed more heterogeneously than the weights of authors’ collaborative links with other authors with whom there are no links in the citation network. Second, in the citation network multistrengths follow a distinct functional behavior depending on the different type of multilink, and are characterized by different 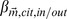 exponents. In fact the fitted values of these exponents are given by 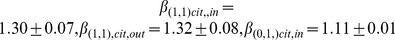, and 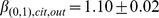. This implies that, on average, highly cited authors are cited by their co-authors to a much greater extent than is the case with poorly cited authors. A similar, though much weaker effect was also found for the citations connecting authors that are not collaborators. Furthermore, in the citation layer the inverse multiparticipation ratio for multilink 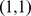 is always larger than the inverse multiparticipation ratio for multilinks 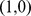 and 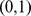 (see Text S1 for details on the statistical test). Finally, when single layers were analyzed separately, we found 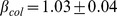 in the collaboration network, and 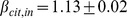 and 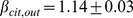 in the citation network. This indicates that in the citation network strength grows super-linearly as a function of degree, i.e., weights are not distributed uniformly. Nevertheless, correlations between weights and types of multilinks cannot be captured if the two individual layers are studied separately.

### 3.1 Assessing the Informational Content of Weighted Multilinks

Recent research on single networks has shown that the entropy of network ensembles provides a very powerful tool for quantifying their complexity [31]–[34]. Here, we propose a theoretical framework based on the entropy of multiplex ensembles for assessing the amount of information encoded in the weighted properties of multilinks. Multiplex weighted network ensembles can be defined as the set of all weighted multiplex networks satisfying a given set of constraints, such as the expected degree sequence and the expected strength sequence in every layer of the multiplex network, or the expected multidegree sequence and the expected multistrength sequence. A set of constraints imposed upon the multiplex network ensemble uniquely determines the probability 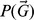 of the multiplex networks in the ensemble (see Materials and Methods). The entropy 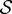 of the multiplex ensemble can be defined in terms of 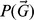 as
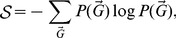
(9)where 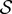 indicates the logarithm of the typical number of multiplex networks in the ensemble. The smaller the entropy, the larger the amount of information stored in the constraints imposed on the network. The entropy can be regarded as an unbiased way to evaluate the informational value of these constraints.

In order to gauge the information encoded in a weighted multiplex network with respect to a null model, we define the indicator 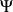, which quantifies how much information is carried by the weight distributions of a weighted multiplex ensemble. In particular, 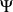 compares the entropy of a weighted multiplex ensemble 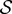 with the entropy of a weighted multiplex ensemble in which the weights are distributed homogeneously. Therefore, 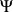 can be defined as
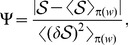
(10)where 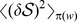 is the standard deviation, and the average 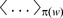 is calculated over multiplex networks with the same structural properties but with weights distributed homogeneously. In particular, when the weight distribution is randomized, the multiplex networks are constrained in such a way that each link must have a minimal weight (i.e., 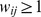), while the remaining of the total weight is distributed randomly over the links. In all the considered network ensembles we have assumed that the weights of the links can only take values that are multiple of a minimal weight. This assumption is by no means a limitation of this approach because for every finite network, there is always a minimal weight in the network such that this hypothesis is verified.

In order to evaluate the amount of information encoded in the weight of links in single layers and compare it to the information supplied by multistrength, we consider the following undirected multiplex ensembles:

- *Correlated weighted multiplex ensemble*. In this ensemble, we fix the expected multidegree sequence 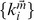, and we set the expected multistrength sequence 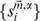 to be
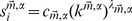
(11)for every layer 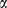. We call 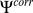 the 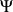 calculated from this ensemble.
- *Uncorrelated weighted multiplex ensemble*. In this ensemble, we set the expected degree 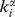 of every node 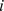 in every layer 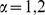 to be equal to the sum of the multidegrees (with 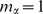) in the correlated weighted multiplex ensemble. We set the expected strengths 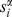 of every node 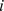 in every layer 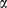 to be equal to the sum of the multistrengths of node 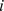 in layer 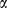 in the correlated weighted multiplex ensemble. We call 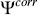 the 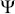 calculated from this ensemble.

In the correlated weighted multiplex ensemble the properties of the multilinks are accounted for, while in the uncorrelated weighted multiplex ensemble the different layers of the multiplex networks are analyzed separately (see Text S1 for the details). Finally, to quantify the additional amount of information carried by the correlated multiplex ensemble with respect to the uncorrelated multiplex ensemble, we define the indicator 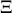 as
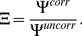
(12)


**Figure 4:**
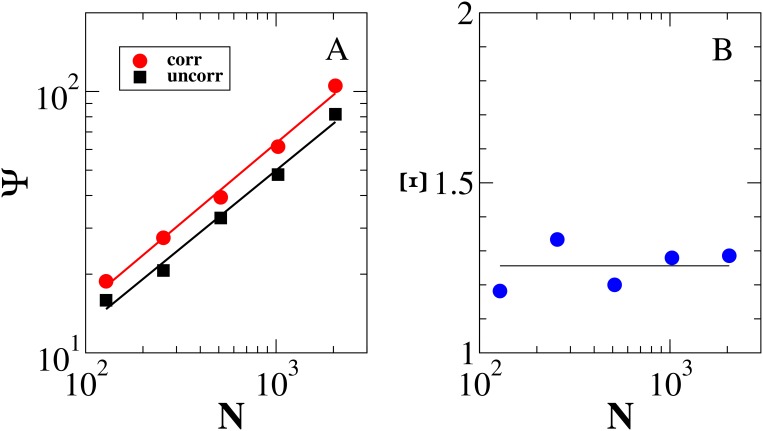
(A) Value of the indicator 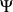 defined in Eq. (10) indicating the amount of information carried by the correlated and the uncorrelated multiplex ensembles of 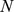 nodes with respect to a null model in which the weights are distributed uniformly over the multiplex network. (B) Value of the indicator 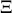 defined in Eq. (12) indicating the additional amount of information encoded in the properties of multilinks in the correlated multiplex ensemble with respect to the corresponding uncorrelated multiplex ensemble. The solid line refers to the average value of 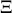 over the different multiplex network sizes.

As an example of a possible application of the indicator 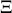, we focus on a case inspired by the CoCi-PRE multiplex network, where we consider different exponents 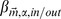 for different multilinks. First, we created the correlated multiplex ensemble with power-law multidegree distributions 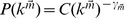 with exponents 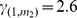 for 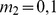 and 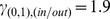 (where for multidegree 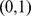 we imposed a structural cut-off). Multistrengths satisfy Eq. (11), with 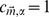 and 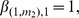 for 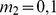; 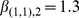, and 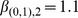. Second, for the second layer, we created the uncorrelated version of the multiplex ensemble which is characterized by a super-linear dependence of the average strength on the degree of the nodes. We then measured 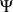 as a function of network size 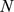 for these different ensembles. Numerically, the average 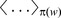 was evaluated from 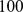 randomizations. Figure 4 shows that 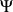 increases with network size 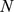 as a power law, and that 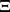 fluctuates around an average value of 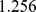. These findings indicate that a significant amount of information is contained in multistrength and cannot be extracted from individual layers separately. Similar results, not shown here, were obtained with a correlated weighted multiplex ensemble characterized by non-trivial inverse multiparticipation ratios.

## Conclusions

In this paper, we have shown that weighted multiplex networks are characterized by significant correlations across layers, and in particular that weights are closely correlated with the multiplex network structure. To properly detect these correlations, we introduced and defined two novel weighted properties of multiplex networks, namely multistrength and the inverse multiparticipation ratio, that cannot be reduced to the properties of single layers. These weighted multiplex properties capture the crucial role played by multilinks in the distribution of weights, i.e., the extent to which there is a link connecting each pair of nodes in every layer of the multiplex network. To illustrate an example of weighted multiplex networks displaying non-trivial correlations between weights and topology, we analyzed the weighted properties of multilinks in two multiplex networks constructed by combining the co-authorship and citation networks involving the authors included in the APS dataset. Finally, based on the entropy of multiplex ensembles, we developed a theoretical framework for evaluating the information encoded in weighted multiplex networks, and proposed the indicator 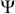 for quantifying the information that can be extracted from a given dataset with respect to a null model in which weights are randomly distributed across links. Moreover, we proposed a new indicator 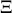 that can be used to evaluate the additional amount of information that the weighted properties of multilinks provide over the information contained in the properties of single layers. In summary, in this paper we have provided compelling evidence that the analysis of multiplex networks cannot be simplified to the partial analysis of single layers, and in particular that non-trivial information can be uncovered only by shifting emphasis on a number of weighted properties of multilinks.

## Materials and Methods

We can build a multiplex ensemble by maximizing the entropy 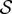 of the ensemble given by Eq. (9) under the condition that the constraints imposed upon the multiplex networks are satisfied on average over the ensemble (soft constraints). We assume there are 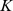 of such constraints determined by the conditions.
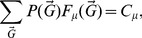
(13)for 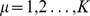, where 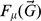 determines one of the structural constraints that we want to impose on average on the multiplex network. The most unbiased multiplex ensemble satisfying the constraints given by Eqs. (13) maximizes the entropy 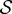 under these constraints. In this ensemble, the probability 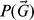 for a multiplex network 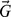 of the ensemble is given by
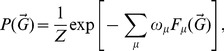
(14)where the normalization constant 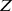 is called the “partition function” of the canonical multiplex ensemble, and is fixed by the normalization condition imposed on 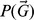, whereas 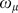 are the Lagrangian multipliers enforcing the constraints in Eq. (13). The values of the Lagrangian multipliers 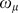 are determined by imposing the constraints given by Eq. (13), while for the probability 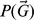 the structural form given by Eq. (14) is assumed. We refer to the entropy 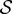 given by Eq. 9 calculated using the probability 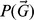 given by Eq. (14) as the Shannon entropy of the multiplex ensemble. For all the details on the derivation of the entropy for these ensembles, we refer the interested reader to the Text S1.

## Supporting Information

Text S1 **Supporting Information Text.**
(PDF)
