# Supplementary material for: Weighted Multiplex Networks
Source: PLoS One. 2014 Jun 6;9(6):e97857. doi: 10.1371/journal.pone.0097857 (PMC4048161; doi:10.1371/journal.pone.0097857)
Supplement: Text S1 — Supporting Information Text. (PDF) [file pone.0097857.s001.pdf]

Supplementary Material:
"Weighted Multiplex networks"

Giulia Menichetti,[1] Daniel Remondini,[1] Pietro Panzarasa,[2] Raúl J. Mondragón,[3] and Ginestra Bianconi[4]

[1] *Department of Physics and Astronomy and INFN Sez. Bologna, Bologna University, Viale B. Pichat 6/2 40127 Bologna, Italy*

[2] *School of Business and Management, Queen Mary University of London, London E1 4NS , United Kingdom*

[3] *School of Electronic Engineering and Computer Science, Queen Mary University of London, London E1 4NS, United Kingdom*

[4] *School of Mathematical Sciences, Queen Mary University of London, London E1 4NS, United Kingdom*

Contents

I. Introduction 1

II. Additional information on the multiplex networks analyzed in this study 1
- A. The two datasets 1
- B. Total overlap and total weighted overlap of the multiplex networks 4
- C. Degree and multidegree distribution of the two multiplex networks 4
- D. Weighted network properties of single layers 6
- E. Statistical analysis of the properties of multilinks in the CoCo-PRL/PRE multiplex network 7
  - 1. Statistical analysis of the average multistrengths in the CoCo-PRL/PRE multiplex network 7
  - 2. Statistical analysis of the average inverse multiparticipation ratio in the CoCo-PRL/PRE multiplex network 8
- F. Statistical analysis of the properties of multilinks in the CoCi-PRE multiplex network 8
  - 1. The statistical analysis of the average multistrengths in the CoCi-PRE multiplex network 10
  - 2. The statistical analysis of the inverse multiparticipation ratio in the CoCi-PRE multiplex network 10

III. Weighted Multiplex Ensembles 11
- A. Definition 11
- B. Canonical weighted multiplex ensembles or exponential weighted multiplex ensembles 11
- C. Uncorrelated and correlated canonical multiplex ensembles 12
- D. Multiplex ensemble with given expected strength sequence and degree sequence in each layer 13
- E. Multiplex ensemble with given expected multidegree sequence and given expected multistrength sequence 14

IV. Background information on Fig. 4 of the main text 15

References 18

I. INTRODUCTION

In this supplementary material we give background information regarding the manuscript 'Weighted Multiplex Networks". The material is organized as follows. In Section II we present additional information about the multiplex datasets analyzed in the manuscript, and provide the details of the statistical analysis of the data. In section III we describe the theory of weighted multiplex ensembles, and in particular we focus on the uncorrelated weighted multiplex ensemble and on the correlated weighted multiplex ensemble used in the main body of the manuscript. Finally, in section IV we provide background information that explains how Fig. 4 in the main text of the manuscript was obtained.

II. ADDITIONAL INFORMATION ON THE MULTIPLEX NETWORKS ANALYZED IN THIS STUDY

A. The two datasets

We have considered the American Physical Society (APS) research data that is organized into two main datasets:

- *Article metadata:* for each article the metadata includes DOI, journal, volume, issue, first page and last page, article id and number of pages, title, authors, affiliations, publication history, PACS codes, table of contents, heading, article type, and copyright information.
- *Citing article pairs:* this dataset consists of pairs of APS articles that cite each other. Each pair is represented by a pair of DOIs. The first id cites the second id.

In the APS metadata an author is usually identified by given name, middle name, and surname. In different articles, the same author can appear with his/her full name or with his/her initials. To deal with this issue, we decided to identify a specific author with the initials of his/her given name and middle name and with his/her full surname. We restricted our analysis to the article metadata and citing article pairs that relate only to PRL and PRE. The total number of PRL articles is $95,516$ and the total number of PRL authors is $117,412$. The total number of PRE articles is $35,944$ and the total number of PRE authors is $36,171$. The number of authors that published both in PRE and PRL is equal to $17,470$.

Among the papers published in PRE and PRL, we focused our study only on those containing a number of authors $n_p \leq 10$. This excludes most of the experimental high-energy collaborations that are typically characterized by a number of authors of a different order of magnitude. We decided to place such a cut-off to the maximum number of authors allowed per paper to avoid biases due to very large publications. Given the cut-off, our study thus becomes limited to $35,766$ PRE articles (99.5 %) and $35,205$ PRE authors (97.3 %) on the one hand, and $89,245$ PRL articles (93.4 %) and $92,436$ PRL authors (78.7 %) on the other. The intersection of these two datasets includes $16,207$ authors (i.e., 92.8 % of the previous intersection).

We analyzed two types of interaction between APS authors: scientific collaborations and citations, with weights defined as follows.

- *Collaborations:* two authors are connected if they co-authored at least one paper. The collaborative interaction between author i and author j is defined as in [1, 2], i.e., the undirected adjacency matrix element a_{ij} is given by

$$a_{ij} = \sum_{p \in I} = \frac{\delta_i^p \delta_j^p}{n_p - 1} \quad i \neq j \tag{1}$$

$$a_{ii} = 0, \tag{2}$$

  where the index p indicates an article in the dataset I, n_p indicates the number of authors of article p and $\delta_i^p = 1$ if node i is an author of article p, and $\delta_i^p = 0$ otherwise. The resulting network is undirected and without self-loops.

- *Citations:* two authors are connected by a directed link if one author cites the other one. In this case, the element a_{ij} of the directed adjacency matrix indicating how many times node i cites node j is given by

$$a_{ij} = \sum_{p,p' \in I} \delta_i^p \delta_j^{p'} b_{p,p'}, \tag{3}$$

  where $b_{p,p'} = 1$ if article p cites article p', and $b_{p,p'} = 0$ otherwise. Moreover δ_i^p is defined as above and indicates whether i is author of article p ($\delta_i^p = 1$) or not ($\delta_i^p = 0$). The resulting network is directed and with self-loops.

We constructed the following two duplex networks:

1. **CoCo-PRL/PRE:** *collaborations among PRL and PRE authors.* The nodes of this multiplex network are the authors who published articles both in PRL and PRE (i.e., $16,207$ authors). These nodes are connected in layer 1 through weighted undirected links indicating the strength of their collaboration in PRL (i.e., co-authorship of PRL articles). The same nodes are connected in layer 2 through weighted undirected links indicating the strength of their collaborations in PRE (i.e., co-authorship of PRE articles).

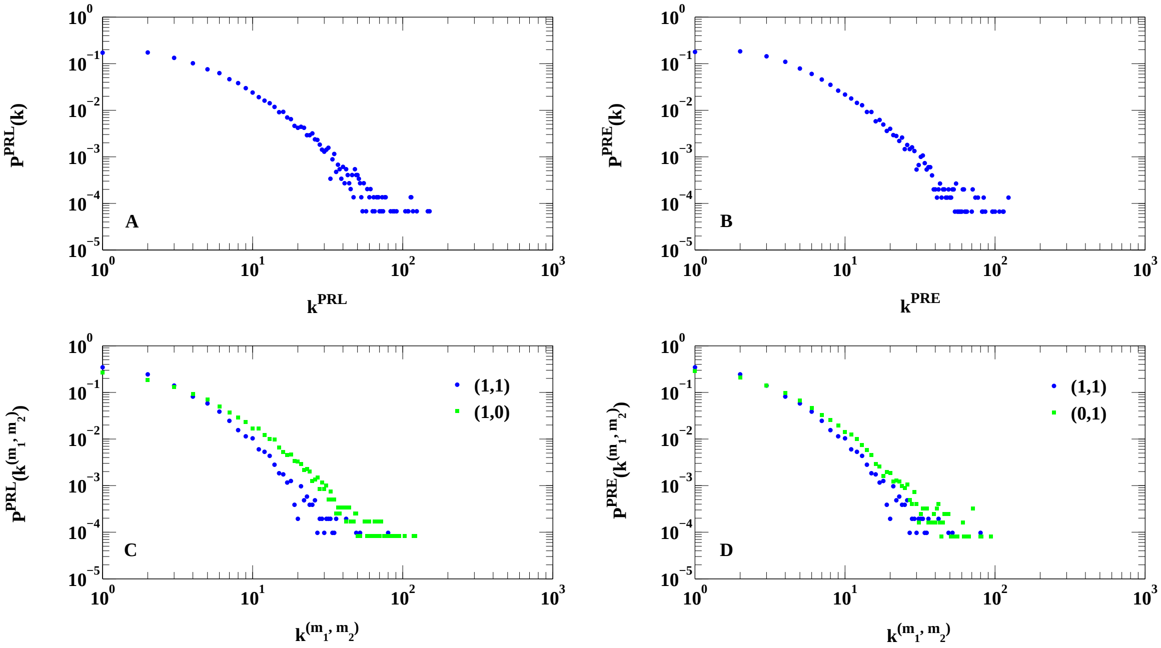

FIG. 1: The degree distributions and the multidegree distributions in the CoCo-PRL/PRE multiplex network.

TABLE I: Total overlap and total weighted overlap in the CoCo-PRL/PRE and CoCi-PRE multiplex networks.

**Dataset**	**Layer**	**Total overlap %**	**Total weighted overlap %**
CoCo-PRL/PRE	PRL	35.75	28.35
CoCo-PRL/PRE	PRE	39.10	33.84
CoCi-PRE	coll	39.51	14.24
CoCi-PRE	cit	12.64	20.76

2. **CoCi-PRE:** *collaborations among PRE authors and citations to PRE articles.* The nodes of this multiplex network are the authors of articles published in PRE (i.e., $35,205$ authors). These nodes are connected in layer 1 through weighted undirected links indicating the strength of their collaboration in PRE (i.e., co-authorship of PRE articles). The same nodes are connected in layer 2 through weighted directed links indicating how many times an author (with articles in PRE) cited another author's work, where citations are limited to those made to PRE articles.

B. Total overlap and total weighted overlap of the multiplex networks

In order to characterize the overlap existing between the links of the multiplex networks, we define the total overlap $O^{\alpha,\alpha'}$ between layer α and layer α' as the total number of pair of nodes (i,j) connected both in layer α and in layer

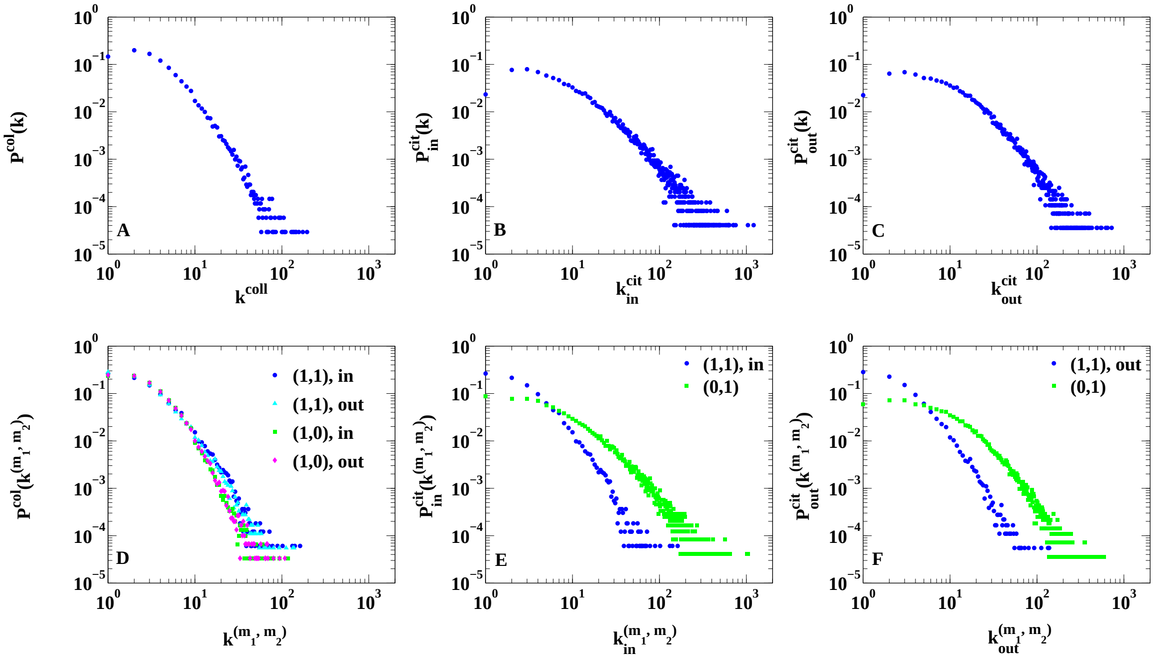

FIG. 2: The degree distributions and the multidegree distributions in the CoCi-PRE multiplex network.

α', i.e.,

$$O^{\alpha,\alpha'} = \sum_{i<j} \theta(a_{ij}^{\alpha})\theta(a_{ij}^{\alpha'}), \tag{4}$$

where $\theta(x) = 1$ if $x > 1$ and $\theta(x) = 0$ otherwise. This definition can be extended to weighted multiplex networks by defining the *total weighted overlap* $O^{(w)\alpha,\alpha'}$ between layer α and layer α' as

$$O^{(w),\alpha,\alpha'} = \sum_{i<j} \min\left(\frac{w_{ij}^{\alpha}}{w_{max}^{\alpha}} \frac{w_{ij}^{\alpha'}}{w_{max}^{\alpha'}}\right), \tag{5}$$

where w_{max}^{α} is the maximal weight in layer α. Table I reports details on the total overlap and total weighted overlap, and indeed shows that our multiplex networks are characterized by a significant overlap of links.

TABLE II: Pearson coefficients r, Spearman coefficients ρ, and Kendall τ coefficients measuring the correlations between the degrees in the different layers and the strengths in the different layers in the CoCi-PRE multiplex network.

r	k_1	k_2^{in}	k_2^{out}	r	s_1	s_2^{in}	s_2^{out}
k_1	1	0.70	0.79	s_1	1	0.80	0.88
k_2^{in}	0.70	1	0.71	s_2^{in}	0.80	1	0.80
k_2^{out}	0.79	0.71	1	s_2^{out}	0.88	0.80	1

ρ	k_1	k_2^{in}	k_2^{out}	ρ	s_1	s_2^{in}	s_2^{out}
k_1	1	0.47	0.49	s_1	1	0.64	0.65
k_2^{in}	0.47	1	0.51	s_2^{in}	0.64	1	0.54
k_2^{out}	0.49	0.51	1	s_2^{out}	0.65	0.54	1

τ	k_1	k_2^{in}	k_2^{out}	τ	s_1	s_2^{in}	s_2^{out}
k_1	1	0.36	0.37	s_1	1	0.53	0.53
k_2^{in}	0.36	1	0.38	s_2^{in}	0.53	1	0.41
k_2^{out}	0.37	0.38	1	s_2^{out}	0.53	0.41	1

TABLE III: Pearson coefficients r, Spearman coefficients ρ, and Kendall τ coefficients measuring the correlations between the degrees in the different layers and the strengths in the different layers in the CoCo-PRL/PRE multiplex network.

r	k_1	k_2
k_1	1	0.74
k_2	0.74	1

r	s_1	s_2
s_1	1	0.65
s_2	0.65	1

ρ	k_1	k_2
k_1	1	0.56
k_2	0.56	1

ρ	s_1	s_2
s_1	1	0.50
s_2	0.50	1

τ	k_1	k_2
k_1	1	0.44
k_2	0.44	1

τ	s_1	s_2
s_1	1	0.37
s_2	0.37	1

C. Degree and multidegree distribution of the two multiplex networks

The nodes $i = 1, 2 \ldots, N$ of the multiplex networks have degrees k_i^1 in layer 1 and k_i^2 in layer 2. Moreover, we can define the multidegree $k_i^{\vec{m}}$ of a generic node i as the sum of the multilinks $\vec{m}$ incident on it. We observe that, since we always have

$$k_i^{\vec{0}} = (N-1) - \sum_{\vec{m} \neq 0} k_i^{\vec{m}}, \tag{6}$$

we can therefore restrict the analysis to multidegrees $\vec{m} \neq \vec{0}$. Figs. 1 and 2 show that the degree and multidegree for both the CoCo-PRL/PRE and the CoCi-PRE multiplex network are broadly distributed. Moreover, in both duplex networks, the degrees each author has in the two layers are positively correlated, as indicated by the Pearson, Spearman, and Kendall correlation coefficients between degrees (See Tables II, III). Finally, also multidegrees in the multiplex networks are correlated, as indicated by their Pearson, Spearman, and Kendall coefficients (See Tables IV, V). These correlations coefficients are calculated both for degree and multidegrees. In what follows, we give the definition in the case of two degree sequences. The extension to multidegree sequences is straightforward.

The Pearson correlation coefficient r between two degree sequences $\{k_i^\alpha\}$ and $\{k_i^\beta\}$ is given by

$$r = \frac{\langle k^\alpha k^\beta \rangle - \langle k^\alpha \rangle \langle k^\beta \rangle}{\sigma_\alpha \sigma_\beta}, \tag{7}$$

where $\sigma_\alpha = \sqrt{\langle k^\alpha k^\alpha \rangle - \langle k^\alpha \rangle^2}$. The Spearman correlation coefficient ρ between the degree sequences $\{k_i^\alpha\}$ and $\{k_i^\beta\}$ in the two layers α and β is given by

$$\rho = \frac{\langle x^\alpha x^\beta \rangle - \langle x^\alpha \rangle \langle x^\beta \rangle}{\sigma(x^\alpha)\sigma(x^\beta)}, \tag{8}$$

where x_i^α is the rank of the degree k_i^α in the sequence $\{k_i^\alpha\}$, and x_i^β is the rank of the degree k_i^β in the sequence $\{k_i^\beta\}$. Moreover, $\sigma(x^\alpha) = \sqrt{\langle (x^\alpha)^2 \rangle - \langle x^\alpha \rangle^2}$, and $\sigma(x^\beta) = \sqrt{\langle (x^\beta)^2 \rangle - \langle x^\beta \rangle^2}$. The Spearman coefficient suffers from the problem that the ranks of the degrees in each degree sequence are not uniquely defined because the degree sequence has in general some degeneracy. Thus, the Spearman correlation coefficient is not a uniquely defined number. The Kendall's τ correlation coefficient between the degree sequences $\{k_i^\alpha\}$ and $\{k_i^\beta\}$ in the two layers α and β is a measure that takes into account the sequence of ranks $\{x_i^\alpha\}$ and $\{x_i^\beta\}$. A pair of nodes i and j are concordant if their ranks have the same order in the two sequences,.i.e., $(x_i^\alpha - x_j^\alpha)(x_i^\beta - x_j^\beta) > 0$; otherwise, they are discordant. The Kendall's τ is defined in terms of the number n_c of concordant pairs and the number n_d of discordant pairs, and is given by

$$\tau = \frac{n_c - n_d}{\sqrt{(n_0 - n_1)(n_0 - n_2)}}, \tag{9}$$

where $n_0 = 1/2N(N-1)$ and the terms n_1 and n_2 account for the degeneracy of the ranks and are given by

$$n_1 = \frac{1}{2} \sum_n u_n(u_n - 1),$$
$$n_2 = \frac{1}{2} \sum_n v_n(v_n - 1), \tag{10}$$

where we call u_n the number of nodes in the nth tied group of the degree sequence $\{k^\alpha\}$, and we call v_n the number of nodes in the nth tied group of the degree sequence $\{k^\beta\}$.

TABLE IV: Pearson correlation coefficient r, Spearman correlation coefficient ρ, and Kendall's τ coefficients measuring the correlations between multidegrees in the CoCi-PRE multiplex network.

r	k_{11}^{in}	k_{11}^{out}	k_{10}^{in}	k_{10}^{out}	k_{01}^{in}	k_{01}^{out}
k_{11}^{in}	1	0.93	0.36	0.52	0.71	0.72
k_{11}^{out}	0.93	1	0.46	0.45	0.67	0.75
k_{10}^{in}	0.36	0.46	1	0.90	0.32	0.48
k_{10}^{out}	0.52	0.45	0.90	1	0.43	0.51
k_{01}^{in}	0.71	0.67	0.32	0.43	1	0.65
k_{01}^{out}	0.72	0.75	0.48	0.51	0.65	1

ρ	k_{11}^{in}	k_{11}^{out}	k_{10}^{in}	k_{10}^{out}	k_{01}^{in}	k_{01}^{out}
k_{11}^{in}	1	0.70	-0.07	0.16	0.66	0.50
k_{11}^{out}	0.70	1	0.15	-0.02	0.48	0.61
k_{10}^{in}	-0.07	0.15	1	0.78	0.05	0.17
k_{10}^{out}	0.16	-0.02	0.78	1	0.18	0.12
k_{01}^{in}	0.66	0.48	0.05	0.18	1	0.45
k_{01}^{out}	0.50	0.61	0.17	0.12	0.45	1

τ	k_{11}^{in}	k_{11}^{out}	k_{10}^{in}	k_{10}^{out}	k_{01}^{in}	k_{01}^{out}
k_{11}^{in}	1	0.63	-0.06	0.13	0.54	0.39
k_{11}^{out}	0.63	1	0.12	-0.02	0.39	0.49
k_{10}^{in}	-0.06	0.12	1	0.68	0.03	0.12
k_{10}^{out}	0.13	-0.02	0.68	1	0.13	0.09
k_{01}^{in}	0.54	0.39	0.03	0.13	1	0.34
k_{01}^{out}	0.39	0.49	0.12	0.09	0.34	1

TABLE V: Pearson coefficients ρ measuring the correlations between multidegrees in the CoCo-PRL/PRE multiplex network.

ρ	k_{11}	k_{10}	k_{01}
k_{11}	1	0.41	0.46
k_{10}	0.41	1	0.53
k_{01}	0.46	0.53	1

ρ	k_{11}	k_{10}	k_{01}
k_{11}	1	0.13	0.13
k_{10}	0.13	1	0.23
k_{01}	0.13	0.23	1

ρ	k_{11}	k_{10}	k_{01}
k_{11}	1	0.17	0.17
k_{10}	0.17	1	0.30
k_{01}	0.17	0.30	1

D. Weighted network properties of single layers

Here we report the weighted network properties of the single layers of our multiplex networks. In general, the average strength s_k^α of nodes with degree k in layer α and the average inverse participation ratio Y_k^α of nodes with degree k in layer α are described by the functional behavior

$$s_k^\alpha \propto k^{\beta_\alpha},$$
$$Y_k^\alpha \propto \frac{1}{k^{\lambda_\alpha}}. \tag{11}$$

We have considered both the CoCo-PRL/PRE dataset and the CoCi-PRE dataset and fitted s_k^α and Y_k^α according to this expected power-law behavior.

TABLE VI: CoCo-PRL/PRE multiplex network: power-law exponents λ_α and β_α determining the functional behavior for the average strength s_k^α of nodes with degree k and the average inverse participation ratio Y_k^α, with α corresponding to the collaboration layer in PRL or in PRE.

layer	α	β_α	R^2	λ_α	R^2
PRL	1	0.96 ± 0.04	0.96	0.84±0.03	0.97
PRE	2	1.01 ± 0.05	0.96	0.80± 0.05	0.94

TABLE VII: CoCi-PRE multiplex network: power-law exponents λ_α and β_α determining the functional behavior for the average strength s_k^α of nodes with degree k and the average inverse participation ratio Y_k^α, with α corresponding to the collaboration layer or to the citation layer, both for PRE. For the citation layer, we consider separately the in-behavior and the out-behavior.

layer	α	β_α	R^2	λ_α	R^2
Co	1	1.03 ± 0.04	0.96	0.79±0.04	0.94
Ci_{in}	2,in	1.13 ± 0.02	0.98	0.72± 0.03	0.85
Ci_{out}	2,out	1.14 ± 0.03	0.97	0.70± 0.04	0.83

The exponents shown in Table VI and Table VII have been computed with the method "regression", function of Matlab [3]. This function performs a multiple linear regression, and for each coefficient gives the 95% confidence interval. In the tables, we show also the coefficient of determination R^2 indicating how well the power-law trend fits the data.

As shown by Fig. 3 and Table VI, the CoCo-PRL/PRE multiplex network is characterized by a linear behavior of average strength as a function of the degree of nodes. Fig. 4 and Table VII show that the CoCi-PRE multiplex network is characterized by a linear behavior of average strength as a function of the degree of nodes in the collaboration network, and by a super-linear behavior in the citation network.

E. Statistical analysis of the properties of multilinks in the CoCo-PRL/PRE multiplex network

In this subsection, we discuss in detail the results of our statistical analysis of the properties of multilinks in the CoCo-PRL/PRE multiplex network. In particular, we focus on the average multistrength of nodes with a given

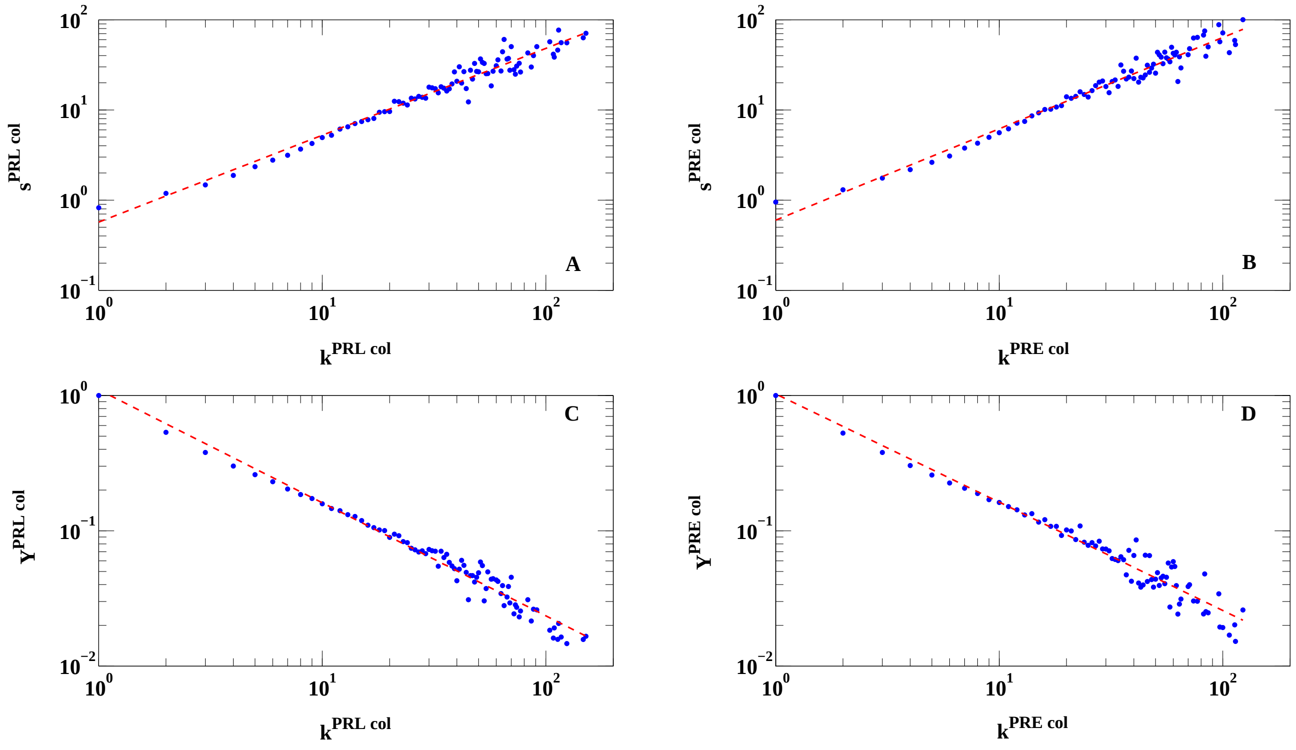

FIG. 3: Average strength versus degree and average inverse participation ratio in the two layers of the CoCo-PRL/PRE multiplex network. The average strength and the average inverse participation ratio follow the functional form described by Eq. (11).

multidegree, i.e., $s^{\vec{m},\alpha}(k^{\vec{m}}) = \left\langle s_i^{\vec{m},\alpha}\delta(k_i^{\vec{m}}, k^{\vec{m}})\right\rangle$, and the average inverse multiparticipation ratio of nodes with a given multidegree, i.e., $Y^{\vec{m},\alpha}(k^{\vec{m}}) = \left\langle Y_i^{\vec{m},\alpha}\delta(k_i^{\vec{m}}, k^{\vec{m}})\right\rangle$. These quantities are expected to scale as

$$\begin{aligned} s^{\vec{m},\alpha}(k^{\vec{m}}) &= e^{q^{\vec{m},\alpha}}(k^{\vec{m}})^{\beta_{\vec{m},\alpha}} \\ Y^{\vec{m},\alpha}(k^{\vec{m}}) &= e^{p^{\vec{m},\alpha}}\frac{1}{(k^{\vec{m}})^{\lambda_{\vec{m},\alpha}}}, \end{aligned} \quad (12)$$

with exponents $\beta_{\vec{m},\alpha} \geq 1$ and $\lambda_{\vec{m},\alpha} \leq 1$. We have computed these exponents with the method "regression", function of Matlab [3] This function performs a multiple linear regression, and for each coefficient gives the 95% confidence interval. We have also computed the coefficient of determination R^2 indicating how well the power-law trend fits the data. For a complete list of the exponents characterizing multistrength and the inverse multiparticipation ratio, see Table VIII. In what follows, we will label the *PRL* collaboration layer as $\alpha = 1$ and the *PRE* collaboration layer as $\alpha = 2$.

1. *Statistical analysis of the average multistrengths in the CoCo-PRL/PRE multiplex network*

In the CoCo-PRL/PRE multiplex network (see Fig. 2 in the main text), the fitted exponents $\beta_{\vec{m},1}$ for multilinks $\vec{m} = (1,1)$ and $\vec{m} = (1,0)$ and the fitted proportionality constants in Eq. (12) are not significantly different. However, we can perform a paired samples Student's t-test to show how the average multistrength per fixed multidegree $s^{\vec{m},\alpha}(k^{\vec{m}})$ is significantly higher for multilinks $(1,1)$ than multilinks $(1,0)$. We have identified pairs of average multistrength $s^{(1,1),1}(k^{(1,1)})$ and $s_k^{(1,0),1}(k^{(1,0)})$, corresponding to the same multidegree value $k^{(1,1)} = k^{(1,0)} = k$. The paired samples Student's t-test returns a test decision for the null hypothesis that the values $\log\left(s^{(1,1),1}(k)/s^{(1,0),1}(k)\right)$ come from a

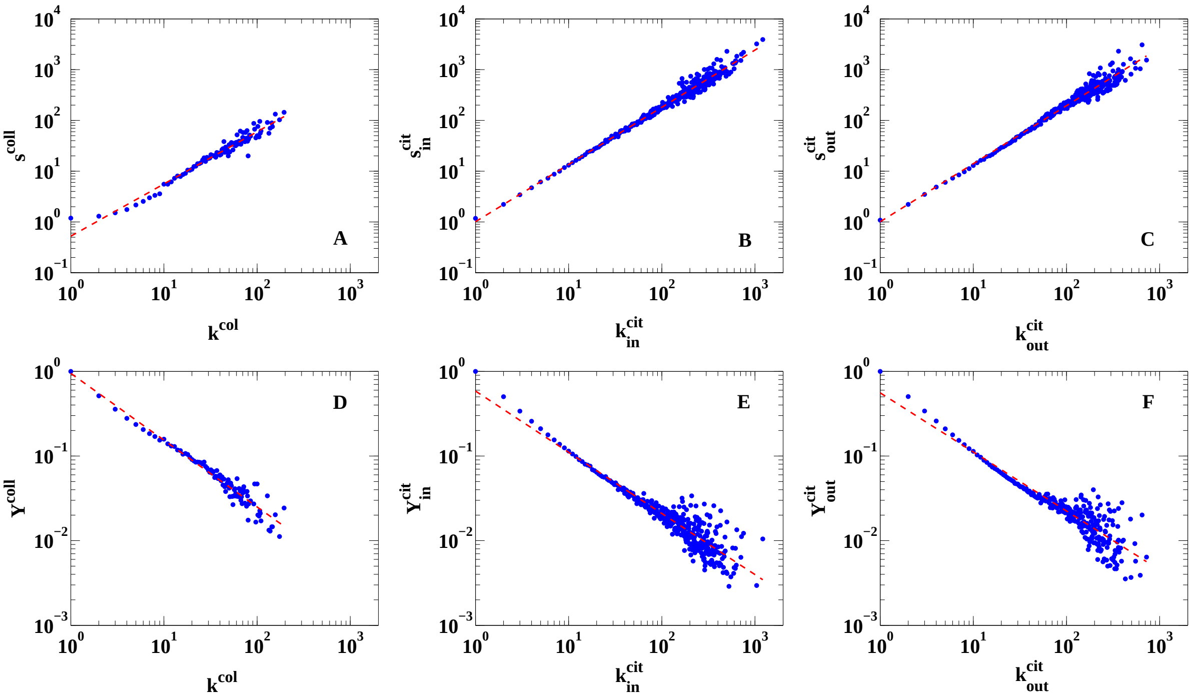

FIG. 4: Average strength and average inverse participation ratio versus degree in the two layers of the CoCi-PRE multiplex network. Average strength and average inverse participation ratio follow the functional form described by Eq. (11).

normal distribution with mean zero and variance from the data. In our case, the null hypothesis is rejected with a p-value equal to $2.90 \cdot 10^{-16}$. Furthermore, $\left\langle \log\left(s^{(1,1),1}(k)/s^{(1,0),1}(k)\right)\right\rangle$ is equal to 0.53. This analysis suggests that for a particular value of degree k the related $s^{(1,1),1}(k)$ is higher than $s^{(1,0),1}(k)$, and the two average multistrengths satisfy the relation $s^{(1,1),1}(k) \approx e^{0.53} s^{(1,0),1}(k)$. Similar results were obtained in the case of the multistrengths on the second layer indicating the collaboration network on *PRE* articles. The null hypothesis is rejected with a p-value equal to $8.98 \cdot 10^{-15}$, and $\left\langle \log\left(s^{(1,1),2}(k)/s^{(0,1),2}(k)\right)\right\rangle$ is equal to 0.57.

2. Statistical analysis of the average inverse multiparticipation ratio in the CoCo-PRL/PRE multiplex network

In the *PRL* layer the fitted exponents $\lambda_{\vec{m},\alpha}$ are significantly different. The weights regarding multilinks $(1,1)$ are distributed more heterogeneously than the weights regarding multilinks $(1,0)$. A similar situation is found also in the *PRE* layer. The paired Student's t-test is also useful to understand the properties of the average inverse multiparticipation ratio. In addition to the fitted exponents, we can perform a t-test as we did previously considering now $Y^{\vec{m},\alpha}(k^{\vec{m}})$. This test underlines how the inverse multiparticipation ratios regarding multilinks (1,1) are significantly higher than those regarding multilinks (1,0) or (0,1). In the case $Y^{(1,1),1}(k)$ vs $Y^{(1,0),1}(k)$, the t-test gives a p-value equal to 0.002 and an average value $\left\langle \log\left(Y^{(1,1),1}(k)/Y^{(1,0),1}(k)\right)\right\rangle = 0.11$. In the case $Y^{(1,1),2}$ vs $Y^{(0,1),2}(k)$, the p-value is equal to $6.64 \cdot 10^{-6}$, and the average value is $\left\langle \log\left(Y^{(1,1),2}(k)/Y^{(0,1),2}(k)\right)\right\rangle = 0.19$.

F. Statistical analysis of the properties of multilinks in the CoCi-PRE multiplex network

We analyzed the average multistrength of nodes with a given multidegree, i.e., $s^{\vec{m},\alpha,(in/out)}(k^{\vec{m}(in/out)}) = \left\langle s_i^{\vec{m},\alpha,(in/out)}\delta(k_i^{\vec{m},(in/out)}, k^{\vec{m},(in/out)})\right\rangle$, and the average inverse multiparticipation ratio of nodes with a given multidegree, i.e., $Y^{\vec{m},\alpha,(in/out)}(k^{\vec{m},(in/out)}) = \left\langle Y_i^{\vec{m},\alpha,(in/out)}\delta(k_i^{\vec{m},(in/out)}, k^{\vec{m},(in/out)})\right\rangle$, where a distinction was made

TABLE VIII: CoCo-PRL/PRE multiplex network: power-law exponents $\lambda_{\vec{m}}$ and $\beta_{\vec{m}}$ and parameters $p_{\vec{m}}$, $q_{\vec{m}}$ determining the functional behavior for average multistrength of nodes with a given multidegree, $s^{\vec{m},\alpha}(k^{\vec{m}})$, and for average inverse multiparticipation ratio of nodes with a given multidegree, $Y^{\vec{m},\alpha}(k^{\vec{m}})$, with α corresponding to the collaboration layer in PRL (1) or in PRE (2). The value of the determination coefficient R^2 for the power-law fits is also reported.

$\vec{m}, \alpha$	$\beta_{\vec{m}}$	$q_{\vec{m}}$	R^2	$\lambda_{\vec{m}}$	$p_{\vec{m}}$	R^2
(1,1), 1	1.06 ± 0.09	-0.51 ± 0.26	0.94	0.74±0.05	-0.09 ± 0.16	0.95
(1,0), 1	0.97 ± 0.03	-0.78 ± 0.10	0.99	0.84± 0.03	0.06 ± 0.12	0.97
(1,1), 2	1.09 ± 0.10	-0.40 ± 0.29	0.93	0.73± 0.06	-0.10 ± 0.19	0.93
(0,1), 2	1.00 ± 0.04	-0.71 ± 0.14	0.98	0.84± 0.05	0.04 ± 0.16	0.95

between incoming and outgoing links in the citation layer. These quantities are expected to scale as

$$\begin{aligned}
s^{\vec{m},1,(in,out)}(k^{m,(\vec{in,out})}) &= e^{q^{m,(\vec{in},out),1}} (k^{\vec{m},(in/out)})^{\beta_{\vec{m},1,(in/out)}} \\
s^{\vec{m},2,(in/out)}(k^{\vec{m},(in/out)}) &= e^{q^{\vec{m},2,(in/out)}} (k^{\vec{m},(in/out)})^{\beta_{\vec{m},2,(in/out)}} \\
Y^{\vec{m},1,(in/out)}(k^{\vec{m},(in/out)}) &= e^{p^{\vec{m},1,(in/out)}} \frac{1}{(k^{\vec{m},(in/out)})^{\lambda_{\vec{m},1,(in/out)}}} \\
Y^{\vec{m},2,(in/out)}(k^{\vec{m},(in/out)}) &= e^{p^{\vec{m},2,(in/out)}} \frac{1}{(k^{\vec{m},(in/out)})^{\lambda_{\vec{m},2,(in/out)}}},
\end{aligned} \tag{13}$$

with exponents $\beta_{\vec{m},\alpha,(in/out)} \geq 1$ and $\lambda_{\vec{m},\alpha,(in/out)} \leq 1$. We have computed these exponents with the method "regression", function of Matlab [3] This function performs a multiple linear regression, and for each coefficient gives the 95% confidence interval. We have also computed the coefficient of determination R^2 indicating how well the power-law trend fits the data. The complete list of the exponents and the multiplication constants characterizing multistrength and the inverse multiparticipation ratio can be found in Table IX together with the correspondening values of R^2. In what follows we will label the *PRE* collaboration layer as $\alpha = 1$ and the *PRE* citation layer as $\alpha = 2$.

1. *The statistical analysis of the average multistrengths in the CoCi-PRE multiplex network*

In the CoCi-PRE multiplex network (see Fig. 3 in the main text), we can perform, at first, a statistical analysis of the multistrengths in the collaboration layer. The fitted exponents $\beta_{(1,1),1,in}$, $\beta_{(1,1),1,out}$, $\beta_{(1,0),1,in}$ and $\beta_{(1,0),1,out}$ are not significantly different. Conversely, the fitted intercepts of the log-log plot, regarding multilinks $(1,1)$, (in/out) are significantly different from the intercept for multilinks $(1,0)$, (in/out).

From a paired samples Student's t-test, in the same way as we did for the average multistrengths in the CoCo-PRL/PRE multiplex network, we obtained that both $s^{(1,1),1,in}(k)$ and $s^{(1,1),1,out}(k)$ are significantly higher than $s^{(1,0),1,in}(k)$ and $s^{(1,0),1,out}(k)$. In the case $s^{(1,1),1,in}(k)$ vs $s^{(1,0),1,in}(k)$, we have a p-value equal to $1.91 \cdot 10^{-35}$ and an average value $\langle \log \left(s^{(1,1),1,in}(k)/s^{(1,0),1,in}(k)\right) \rangle = 0.78$. In the case $s^{(1,1),1,out}(k)$ vs $s^{(1,0),1,out}(k)$, we have a p-value equal to $9.93 \cdot 10^{-30}$ and an average value $\langle \log \left(s^{(1,1),1,out}(k)/s^{(1,0),1,out}(k)\right) \rangle = 0.80$.

Based on the fitted parameters and the Student's t-test, the data suggest that both multidegrees for multilinks $(1,1)$ and multilinks $(1,0)$ have a linear relation with their own multistrengths in the collaboration layer, and that multistrengths (1,1) are related to multistrengths (1,0) by a multiplicative constant. In the citation layer, the fitted exponents $\beta_{\vec{m},in/out}$ indicate a super-linear scaling, and are significantly different (see Table IX).

2. The statistical analysis of the inverse multiparticipation ratio in the CoCi-PRE multiplex network

For the collaboration layer, comparing $\lambda_{(1,1),1,in}$ with $\lambda_{(1,0),1,in}$, and $\lambda_{(1,1),1,out}$ with $\lambda_{(1,0),1,out}$, the confidence intervals of these fitted exponents do not overlap for a narrow window. Performing the t-test as usual, we found that the inverse multiparticipation ratio for multilinks $(1,1)$ is always larger than the inverse multiparticipation ratio for multilinks $(1,0)$. In the case $Y^{(1,1),1,in}(k)$ vs $Y^{(1,0),1,in}(k)$, the t-test gives a p-value equal to $3.70 \cdot 10^{-17}$ and an average value $\left\langle \log\left(Y^{(1,1),1,in}(k)/Y^{(1,0),1,in}(k)\right)\right\rangle = 0.39$. In the case $Y^{(1,1),1,out}$ vs $Y^{(1,0),1,out}(k)$, the p-value is equal to $5.48 \cdot 10^{-19}$ and the average value is $\left\langle \log\left(Y^{(1,1),1,out}(k)/Y^{(1,0),1,out}(k)\right)\right\rangle = 0.33$.

In the *in*− and *out*−citation layers, the fitted exponents $\lambda_{\vec{m},2,(in/out)}$ regarding multilinks $(1,1)$ are not significantly different from those regarding multilinks $(0,1)$. Nevertheless, the paired Student's t-test shows how the inverse multiparticipation ratio for multilinks $(1,1)$ is always larger than the inverse multiparticipation ratio for multilinks $(0,1)$. In the case $Y^{(1,1),2,in}(k)$ vs $Y^{(0,1),2,in}(k)$, the t-test gives a p-value equal to $7.60 \cdot 10^{-21}$ and an average value $\left\langle \log\left(Y^{(1,1),2,in}(k)/Y^{(0,1),2,in}(k)\right)\right\rangle = 0.34$. In the case $Y^{(1,1),2,out}(k)$ vs $Y^{(0,1),2,out}(k)$, the p-value is equal to $1.12 \cdot 10^{-15}$ and the average value is $\left\langle \log\left(Y^{(1,1),2,out}(k)/Y^{(0,1),2,out}(k)\right)\right\rangle = 0.34$.

TABLE IX: CoCi-PRE multiplex network: power-law exponents λ_α and β_α and parameters $p_{\vec{m}}$, $q_{\vec{m}}$ determining the functional behavior for the average multistrength of nodes with a given multidegree $s^{\vec{m},\alpha,(in/out)}(k^{\vec{m}(in/out)})$ and the average inverse multiparticipation ratio of nodes with a given multidegree, $Y^{\vec{m},\alpha,(in/out)}(k^{\vec{m},(in,out)})$, with α corresponding to the collaboration layer (1) or to the citation layer (2), both for PRE. The coefficient of determination R^2 determining the quality of the power-law fit is also reported

$\vec{m}, \alpha$, in/out	$\beta_{\vec{m}}$	$q_{\vec{m}}$	R^2	$\lambda_{\vec{m}}$	$p_{\vec{m}}$	R^2
(1,1), 1, in	1.02 ± 0.04	-0.33 ± 0.15	0.97	0.76±0.05	-0.03 ± 0.16	0.94
(1,1), 1, out	1.05 ± 0.04	-0.38 ± 0.14	0.98	0.77±0.05	-0.03 ± 0.17	0.94
(1,0), 1, in	0.98 ± 0.05	-0.97 ± 0.17	0.96	0.88± 0.03	-0.03 ± 0.09	0.99
(1,0), 1, out	0.97 ± 0.05	-0.95 ± 0.16	0.97	0.90± 0.02	0.00 ± 0.06	0.99
(1,1), 2, in	1.30 ± 0.07	0.47 ± 0.25	0.95	0.73± 0.05	-0.17 ± 0.16	0.94
(1,1), 2, out	1.32 ± 0.08	0.45 ± 0.26	0.95	0.74± 0.04	-0.50 ± 0.20	0.80
(0,1), 2, in	1.11 ± 0.01	-0.01 ± 0.07	0.99	0.75± 0.05	-0.12 ± 0.16	0.95
(0,1), 2, out	1.10 ± 0.02	0.06 ± 0.09	0.98	0.69± 0.05	-0.62 ± 0.22	0.77

III. WEIGHTED MULTIPLEX ENSEMBLES

A. Definition

A weighted multiplex network is formed by N nodes connected within M weighted networks $G_\alpha = (V, E_\alpha)$, with $\alpha = 1, \ldots, M$ and $|V| = N$. Therefore we can represent a multiplex network as $\vec{G} = (G_1, G_2, \ldots, G_\alpha, \ldots G_M)$. Each network G_α is fully described by the adjacency matrix of elements a_{ij}^α, with $a_{ij}^\alpha = w_{ij}^\alpha > 0$ if there is a link of weight w_{ij}^α between nodes i and j in layer α, and $a_{ij}^\alpha = 0$ otherwise. In what follows, in order to simplify the treatment of the weighted multiplex networks, we will assume that the weight of the link between any pair of nodes i and j, $a_{ij}^\alpha = w_{ij}^\alpha$, can only take integer values. This is not a major limitation because in a large number of weighted multiplex networks the weights of the links can be considered as multiples of a minimal weight.

B. Canonical weighted multiplex ensembles or exponential weighted multiplex ensembles

The canonical network ensembles (also known as exponential random graphs) are a very powerful tool for building null models of networks [4, 5]. Here we generalize the formalism developed for unweighted multiplex ensembles [6] to take weighted multiplex ensembles into account.

The construction of the canonical weighted multiplex ensembles or exponential random multiplex graphs follows closely the derivation or the exponential random graphs. A weighted multiplex ensemble is defined once the probability $P(\vec{G})$ of any possible weighted multiplex is given. We can build a canonical multiplex ensemble by maximizing the entropy $\mathcal{S}$ of the ensemble given by

$$\mathcal{S} = -\sum_{\vec{G}} P(\vec{G}) \log P(\vec{G}), \tag{14}$$

under the condition that the soft constraints we want to impose are satisfied. We assume there are K of such constraints determined by the conditions

$$\sum_{\vec{G}} P(\vec{G}) F_\mu(\vec{G}) = C_\mu, \tag{15}$$

for $\mu = 1, 2 \ldots, K$, where $F_\mu(\vec{G})$ determines one of the structural constraints that we want to impose on the multiplex network. Therefore, the maximal-entropy multiplex ensemble satisfying the constraints given by Eqs. (15) is the solution of the following system of equations

$$\frac{\partial}{\partial P(\vec{G})} \left[\mathcal{S} - \sum_{\mu=1}^{K} \omega_\mu \sum_{\vec{G}} F_\mu(\vec{G}) P(\vec{G}) - \Lambda \sum_{\vec{G}} P(\vec{G}) \right] = 0, \tag{16}$$

where the Lagrangian multiplier Λ enforces the normalization of the $P(\vec{G})$ probability distribution, and the Lagrangian multiplier ω_μ enforces the constraint μ. Therefore, we obtain that the probability of a multiplex network $P(\vec{G})$ in a canonical multiplex ensemble is given by

$$P(\vec{G}) = \frac{1}{Z} \exp\left[-\sum_\mu \omega_\mu F_\mu(\vec{G}) \right], \tag{17}$$

where the normalization constant $Z = \exp(1 + \Lambda)$ is called the "partition function" of the canonical multiplex ensemble and is fixed by the normalization condition on $P(\vec{G})$. Thus, Z is given by

$$Z = \sum_{\vec{G}} \exp\left[-\sum_\mu \omega_\mu F_\mu(\vec{G}) \right]. \tag{18}$$

The values of the Lagrangian multipliers ω_μ are determined by imposing the constraints given by Eq. (15) assuming for the probability $P(\vec{G})$ the structural form given by Eq. (17). From the definition of the partition function Z and

Eq. 17, it can easily be shown that the Lagrangian multipliers ω_μ can be expressed as the solutions of the following set of equations

$$C_\mu = -\frac{\partial \log Z}{\partial \omega_\mu}. \tag{19}$$

In this ensemble, we can then relate the entropy $\mathcal{S}$ (given by Eq. (14)) to the canonical partition function Z, and we obtain

$$\mathcal{S} \;=\; \sum_\mu \omega_\mu C_\mu + \log Z. \tag{20}$$

We call the entropy $\mathcal{S}$ of the canonical multiplex ensemble the *Shannon entropy* of the ensemble.

C. Uncorrelated and correlated canonical multiplex ensembles

Multiplex ensembles can be distinguished between uncorrelated and correlated ones [6]. For uncorrelated multiplex ensembles, the probability of a multiplex network $P(\vec{G})$ is factorizable into the probability $P_\alpha(G_\alpha)$ of each single network G_α in layer α, i.e.,

$$P(\vec{G}) = \prod_{\alpha=1}^{M} P_\alpha(G_\alpha). \tag{21}$$

Therefore, the entropy $\mathcal{S}$ of any uncorrelated multiplex ensemble given by Eq. (14) with $P(\vec{G})$ given by Eq. (21) is additive in the number of layers, i.e.,

$$\mathcal{S} = \sum_{\alpha=1}^{M} \mathcal{S}_\alpha = -\sum_{\alpha=1}^{M} P_\alpha(G_\alpha)\log P_\alpha(G_\alpha). \tag{22}$$

For a canonical uncorrelated multiplex ensemble, $P(\vec{G})$ has to satisfy both Eq. (21) and Eq. (17). Therefore, in order to have an uncorrelated multiplex ensemble, the functions $F_\mu(\vec{G})$ should be equal to a linear combination of constraints $f_{\mu,\alpha}(G_\alpha)$ on the networks G_α in a single layer α, i.e.,

$$F_\mu(\vec{G}) = \sum_{\alpha}^{M} f_{\mu,\alpha}(G_\alpha). \tag{23}$$

A special case of this type of constraints is given when each constraint depends on a single network G_α in layer α. An example of this type of constraints will be discussed in the following subsection where we will focus on the important case in which the constraints are the strength sequence $\{s_i^\alpha\}$ in any layer α, and the degree sequence $\{k_i^\alpha\}$ in any layer α.

Moreover, we can define the marginal probability for a specific value of the element a_{ij}^α

$$\pi_{ij}^\alpha(a_{ij}^\alpha = w) = \sum_{\vec{G}} P(\vec{G})\delta(a_{ij}^\alpha, w), \tag{24}$$

where $\delta(x,y)$ is the Kronecker delta. The marginal probabilities $\pi_{ij}^\alpha(a_{ij}^\alpha)$ sum up to one

$$\sum_{a_{ij}^\alpha=0}^{\infty} \pi_{ij}(a_{ij}^\alpha) = 1. \tag{25}$$

We can also calculate the average weight $\langle a_{ij}^\alpha\rangle$ of links between nodes i and j as

$$\langle a_{ij}^\alpha\rangle = \sum_{\vec{G}} P(\vec{G}) a_{ij}^\alpha = \sum_{a_{ij}^\alpha=0}^{\infty} a_{ij}^\alpha \pi_{ij}(a_{ij}^\alpha). \tag{26}$$

In layer α, a link between two nodes i and j exists with probability p_{ij}^α, related with all the possible weights different from zero

$$p_{ij}^\alpha = \sum_{\vec{G}} P(\vec{G})\theta(a_{ij}^\alpha) = \sum_{a_{ij}^\alpha\neq 0}^{\infty} \pi_{ij}^\alpha(a_{ij}^\alpha). \tag{27}$$

D. Multiplex ensemble with given expected strength sequence and degree sequence in each layer

Here we consider the relevant example of the uncorrelated multiplex ensemble in which we fix the expected strength s_i^α and the expected degree k_i^α of every node i in each layer α. We have $K = M \cdot 2N$ constraints in the system. These constraints are given by

$$\begin{aligned}\sum_{\vec{G}} F_{i,\alpha}(\vec{G})P(\vec{G}) &= \sum_{\vec{G}} \left(\sum_{j\neq i} a_{ij}^\alpha\right) P(\vec{G}) = s_i^\alpha \\ \sum_{\vec{G}} F_{i,\alpha}(\vec{G})P(\vec{G}) &= \sum_{\vec{G}} \left(\sum_{j\neq i} \theta(a_{ij}^\alpha)\right) P(\vec{G}) = k_i^\alpha,\end{aligned} \tag{28}$$

with $\alpha = 1, 2, \ldots, M$. We introduce the Lagrangian multipliers $w_{i,\alpha}$ for the first set of $N \cdot M$ constraints and the Lagrangian multipliers $\omega_{i,\alpha}$ for the second set of $N \cdot M$ constraints. Therefore, the probability $P(\vec{G})$ of a multiplex network in this ensemble, in general given by Eq. (17), in this specific example is given by

$$P(\vec{G}) = \frac{1}{Z} \exp\left[-\sum_{\alpha=1}^{M}\sum_{i=1}^{N} w_{i,\alpha} \sum_{j\neq i} a_{ij}^\alpha - \sum_{\alpha=1}^{M}\sum_{i=1}^{N} \omega_{i,\alpha} \sum_{j\neq i} \theta(a_{ij}^\alpha)\right],$$

where the partition function Z can be expressed explicitly as

$$\begin{aligned} Z &= \sum_{\vec{G}} \exp\left[-\sum_{\alpha=1}^{M}\sum_{i=1}^{N}\sum_{j\neq i}\left(w_{i,\alpha}a_{ij}^\alpha + \omega_{i,\alpha}\theta(a_{ij}^\alpha)\right)\right] \\ &= \prod_{\alpha=1}^{M}\prod_{i<j}\left(1 + \frac{e^{-(\omega_{i,\alpha}+\omega_{j,\alpha})-(w_{i,\alpha}+w_{j,\alpha})}}{1-e^{-(w_{i,\alpha}+w_{j,\alpha})}}\right),\end{aligned} \tag{29}$$

and the Lagrangian multipliers are fixed by the conditions Eqs. (28). From Eq. (24) we write the marginal probabilities $\pi_{ij}^\alpha(a_{ij}^\alpha)$ for this specific ensemble that are given by

$$\pi_{ij}^\alpha(a_{ij}^\alpha) = \frac{e^{-(w_{i,\alpha}+w_{j,\alpha})a_{ij}^\alpha - (\omega_{i,\alpha}+\omega_{j,\alpha})\theta(a_{ij}^\alpha)}\left(1-e^{-(w_{i,\alpha}+w_{j,\alpha})}\right)}{1+e^{-(w_{i,\alpha}+w_{j,\alpha})}\left(e^{-(\omega_{i,\alpha}+\omega_{j,\alpha})}-1\right)}. \tag{30}$$

The average weight of the link (i, j) in layer α, i.e., $\langle a_{ij}^\alpha\rangle$, is given by Eq. (26) that in this case reads

$$\langle a_{ij}^\alpha\rangle = = \frac{e^{-(\omega_{i,\alpha}+\omega_{j,\alpha})+(w_{i,\alpha}+w_{j,\alpha})}}{(e^{w_{i,\alpha}+w_{j,\alpha}}-1)(e^{-(\omega_{i,\alpha}+\omega_{j,\alpha})}+e^{w_{i,\alpha}+w_{j,\alpha}}-1)}. \tag{31}$$

Moreover, from Eq. (27) the probability p_{ij}^α that the link (i, j) in layer α has weight different from zero is given by

$$p_{ij}^\alpha = = \frac{e^{-(\omega_{i,\alpha}+\omega_{j,\alpha})}}{e^{-(\omega_{i,\alpha}+\omega_{j,\alpha})}+e^{w_{i,\alpha}+w_{j,\alpha}}-1}. \tag{32}$$

Finally, the probability of a multiplex network $\vec{G}$ in this ensemble, characterized by the M adjacency matrices $\mathbf{a}^\alpha$, is given by

$$P(\vec{G}) = \prod_{\alpha=1}^{M}\prod_{i<j}\pi_{ij}(a_{ij}^\alpha), \tag{33}$$

with the marginals $\pi_{ij}^\alpha(a_{ij}^\alpha)$ given by Eq. (30).

Therefore the entropy $\mathcal{S}$ of this canonical multiplex ensemble is given by

$$\mathcal{S} = -\sum_{\alpha=1}^{M}\sum_{i<j}\sum_{a_{ij}^\alpha=0}^{\infty}\pi_{ij}^\alpha(a_{ij}^\alpha)\log(\pi_{ij}^\alpha(a_{ij}^\alpha)), \tag{34}$$

with the marginals $\pi_{ij}^\alpha(a_{ij}^\alpha)$ given by Eqs. (30).

E. Multiplex ensemble with given expected multidegree sequence and given expected multistrength sequence

Here we consider the example of the correlated weighted multiplex ensemble, in which we fix the expected multidegrees $k_i^{\vec{m}}$ of node i, for each node $i = 1, \ldots, N$, for each $\vec{m}$. Moreover, in addition to these constraints we impose also a given expected strength $s_i^{\vec{m},\alpha}$ for each node $i = 1, 2, \ldots, N$ and each multilink $\vec{m}$, in each layer α where $m_\alpha = 1$. The number of constraints is therefore $K = 2^M \cdot N + (2^{M-1}) \cdot M \cdot N$. In particular, the constraints we are imposing are

$$\sum_{\vec{G}} F_i^{\vec{m}}(\vec{G}) P(\vec{G}) \quad = \sum_{\vec{G}} \left(\sum_{j \neq i} A_{ij}^{\vec{m}} \right) P(\vec{G}) = k_i^{\vec{m}} \tag{35}$$

$$\sum_{\vec{G}} F_{i,\alpha}^{\vec{m}}(\vec{G}) P(\vec{G}) \; = \sum_{\vec{G}} \left(\sum_{j \neq i} A_{ij}^{\vec{m}} a_{ij}^{\alpha} \right) P(\vec{G}) = s_{i,\alpha}^{\vec{m}}, \tag{36}$$

where we have now used the multiadjacency matrices $A_{ij}^{\vec{m}}$ with elements given by

$$A_{ij}^{\vec{m}} = \prod_{\alpha=1}^{M} \left\{ \theta(a_{ij}^{\alpha}) m_\alpha + [1 - \theta(a_{ij}^{\alpha})](1 - m_\alpha) \right\}. \tag{37}$$

Here we introduce the Lagrangian multipliers $\omega_i^{\vec{m}}$ for the first set of constraints and the Lagrangian multipliers $w_{i,\alpha}^{\vec{m}}$ for the second set of constraints. Without loss of generality, if $m_\alpha = 0$ we set $w_{\alpha}^{\vec{m}} = 1/2$. We can do this because the probability of a multiplex network does not depend on any of these values, and we need to define this Lagrangian multipliers only for simplifying the notation. Using these expression for the Lagrangian multipliers, we obtain the following expression for the probability $P(\vec{G})$ of the multiplex network in the ensembles

$$\begin{aligned} P(\vec{G}) &= \frac{1}{Z} \exp \left[- \sum_{\vec{m}} \sum_{i=1}^{N} \sum_{j \neq i} \left(\omega_i^{\vec{m}} A_{ij}^{\vec{m}} + \sum_{\alpha=1}^{M} w_{i,\alpha}^{\vec{m}} A_{ij}^{\vec{m}} a_{ij}^{\alpha} \right) \right] \\ &= \frac{1}{Z} \exp \left[- \sum_{i<j} \sum_{\vec{m}} (\omega_i^{\vec{m}} + \omega_j^{\vec{m}}) A_{ij}^{\vec{m}} - \sum_{i<j} \sum_{\vec{m}} \sum_{\alpha=1}^{M} (w_{i,\alpha}^{\vec{m}} + w_{j,\alpha}^{\vec{m}}) A_{ij}^{\vec{m}} a_{ij}^{\alpha} \right]. \end{aligned} \tag{38}$$

The partition function Z can be expressed explicitly as

$$Z = \prod_{i<j} \mathcal{Z}_{ij}, \tag{39}$$

where $\mathcal{Z}_{ij}$ is given by

$$\mathcal{Z}_{ij} = \sum_{\vec{m}} e^{-(\omega_i^{\vec{m}} + \omega_j^{\vec{m}})} \prod_{\alpha=1}^{M} \left(\frac{e^{-(w_{i,\alpha}^{\vec{m}} + w_{j,\alpha}^{\vec{m}})}}{1 - e^{-(w_{i,\alpha}^{\vec{m}} + w_{j,\alpha}^{\vec{m}})}} \right)^{m_\alpha}. \tag{40}$$

Finally, the Lagrangian multipliers are fixed by the conditions given by Eqs. (36).

We now indicate with $\vec{a}_{ij}$ the vector $(a_{ij}^1, a_{ij}^2, \ldots, a_{ij}^{\alpha}, \ldots, a_{ij}^{M})$. The probability of a multiplex network $P(\vec{G})$ can be rewritten as

$$P(\vec{G}) = \prod_{i<j} \pi_{ij}(\vec{a}_{ij}), \tag{41}$$

where the probability of a specific multiweight $\vec{a}_{ij}$ between nodes i and j is

$$\pi_{ij}(\vec{a}_{ij}) = \frac{e^{-(\omega_i^{\vec{m}^{ij}} + \omega_j^{\vec{m}^{ij}})} \prod_{\alpha=1}^{M} \left(e^{-(w_{i,\alpha}^{\vec{m}^{ij}} + w_{j,\alpha}^{\vec{m}^{ij}}) a_{ij}^{\alpha}} \right)^{m_\alpha^{ij}}}{\mathcal{Z}_{ij}}, \tag{42}$$

where $\vec{m}^{ij} = (m_1^{ij}, \ldots, m_\alpha^{ij}, \ldots, m_m^{ij})$ with $m_\alpha^{ij} = \theta(a_{ij}^{\alpha})$. We note here that $\pi_{ij}(\vec{a}_{ij})$ satisfies the following normalization condition

$$\sum_{\vec{a}_{ij}} \pi_{ij}(\vec{a}_{ij}) = 1. \tag{43}$$

The average weight $\langle a_{ij}^{\alpha}A_{ij}^{\vec{m}}\rangle$ of multilink $\vec{m}$ between nodes i and j in layer α and the probability $p_{ij}^{\vec{m}}$ of multilink $\vec{m}$ between nodes i and j are given by

$$\langle a_{ij}^{\alpha}A_{ij}^{\vec{m}}\rangle = \frac{e^{-(\omega_i^{\vec{m}}+\omega_j^{\vec{m}})}}{\mathcal{Z}_{ij}}\left(\frac{1}{1-e^{-(w_{i,\alpha}^{\vec{m}}+w_{j,\alpha}^{\vec{m}})}}\right)\prod_{\beta=1}^{M}\left(\frac{e^{-(w_{i,\beta}^{\vec{m}}+w_{j,\beta}^{\vec{m}})}}{1-e^{-(w_{i,\beta}^{\vec{m}}+w_{j,\beta}^{\vec{m}})}}\right)^{m_\beta}, \tag{44}$$

$$p_{ij}^{\vec{m}} = \frac{e^{-(\omega_i^{\vec{m}}+\omega_j^{\vec{m}})}}{\mathcal{Z}_{ij}}\prod_{\alpha=1}^{M}\left(\frac{e^{-(w_{i,\alpha}^{\vec{m}}+w_{j,\alpha}^{\vec{m}})}}{1-e^{-(w_{i,\alpha}^{\vec{m}}+w_{j,\alpha}^{\vec{m}})}}\right)^{m_\alpha}. \tag{45}$$

Finally, since the probability of a multiplex network $P(\vec{G})$ is given by Eq. (41), the entropy $\mathcal{S}$ defined in Eq. (14) in this ensemble is given by

$$\mathcal{S} = -\sum_{i<j}\sum_{\vec{a}_{ij}}\pi_{ij}(\vec{a}_{ij})\log\pi_{ij}(\vec{a}_{ij}). \tag{46}$$

IV. BACKGROUND INFORMATION ON FIG. 4 OF THE MAIN TEXT

As an example of a possible application of the indicators Ψ and Ξ, we analyze a case inspired by the CoCi-PRE multiplex network. Due to the numerical limitations of the programs that are able to evaluate the entropy of multiplex ensembles, we perform a finite-size analysis of the indicators Ψ and Ξ as a function of the size of the multiplex network $N = 128, 256, \ldots, 2048$. In particular, we consider the following undirected multiplex ensembles:

- *Correlated weighted multiplex ensemble.* First, we create the correlated multiplex ensemble with power-law expected multidegree distributions with exponents $\gamma^{(1,m_2)} = 2.6$ for $m_2 = 0,1$ and $\gamma^{(0,1)} = 1.9$ (for multidegree $(0,1)$ we impose a structural cut-off). In particular, in order to avoid the effects of fluctuations in the expected multidegree sequence, we rank the multidegrees as $r = 1,2,\ldots N$, and take the sequence in which the multidegree $k_r^{\vec{m}}$ of rank r is defined by

$$\frac{r}{N} = \int_{k_r^{\vec{m}}}^{K} P(k^{\vec{m}})dk^{\vec{m}}, \tag{47}$$

  where we take the maximal cut-off $K = N^{1/\gamma^{\vec{m}}}$ for $\gamma^{\vec{m}} > 2$ and $K = \sqrt{\langle k^{\vec{m}}\rangle N}$ for $\gamma^{\vec{m}} < 2$. Note that this is possible because the expected multidegrees are real values. Moreover, the expected multistrengths are assumed to satisfy

$$s_i^{\vec{m},\alpha} = c_{\vec{m},1}(k^{\vec{m},\alpha})^{\lambda_{\vec{m},\alpha}}, \tag{48}$$

  with $c_{\vec{m},\alpha} = 1$, $\beta^{(1,m_2),1} = 1$ for $m_2 = 0,1$, $\beta^{(1,1),2} = 1.3$, and $\beta^{(0,1),2} = 1.1$.

- *Uncorrelated weighted multiplex ensemble.* In this ensemble, we set the expected degree k_i^{α} of every node i in every layer $\alpha = 1,2$ to be equal to the sum of the expected multidegrees (with $m_\alpha = 1$) in the correlated weighted multiplex ensemble. Moreover, we set the expected strengths s_i^{α} of every node i in every layer α to be equal to the sum of the expected multistrengths of node i in layer α in the correlated weighted multiplex ensemble.

We measure the indicator Ψ that compares the entropy of a weighted multiplex ensemble $\mathcal{S}$ with the entropy of a weighted multiplex ensemble in which weights are distributed homogeneously. Therefore, Ψ can be defined as

$$\Psi = \frac{|\mathcal{S} - \langle\mathcal{S}\rangle_{\pi(w)}|}{\langle(\delta\mathcal{S})^2\rangle_{\pi(w)}}, \tag{49}$$

where the average $\langle\ldots\rangle_{\pi(w)}$ is calculated over multiplex networks with the same structural properties but with weights distributed homogeneously. In particular, when the weight distribution is randomized, the multiplex networks are constrained in such a way that each link must have a minimal weight (i.e. $w_{ij} > 1$), while the remaining of the total weight is distributed randomly across links. When numerically evaluating $\langle\ldots\rangle_{\pi(w)}$, we obtain the average over 100 weight randomizations.

The distribution $P(\mathcal{S})$ of the entropy $\mathcal{S}$ calculated over these randomizations, both for the uncorrelated weighted multiplex ensemble and for the correlated weighted multiplex ensemble, is shown in Fig. 5. In both cases, we observe

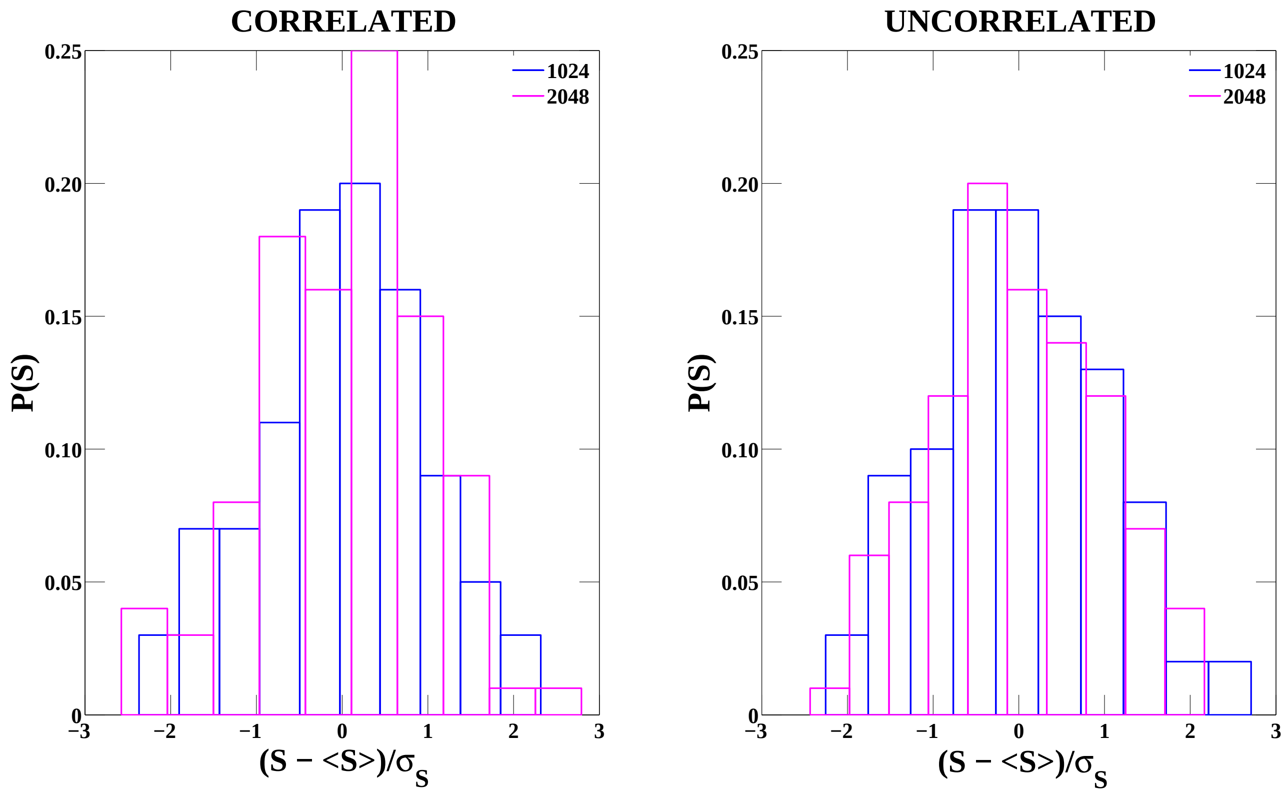

FIG. 5: The $P(\mathcal{S})$ distribution in the null models for correlated and uncorrelated multiplex ensembles in which the weights are distributed uniformly over the links of the multiplex network. The $P(S)$ distributions are calculated over 100 randomizations of the weights for multiplex networks of $N = 1024$ and $N = 2048$ nodes.

a distribution that can be fitted by a Gaussian function with mean and variance scaling as $\langle \mathcal{S} \rangle \propto N \log N$ and $\left\langle (\delta \mathcal{S})^2 \right\rangle_{\pi(w)} \propto \sqrt{N}$ (See Fig. 6). We call Ψ^{corr} the indicator Ψ calculated on the correlated multiplex ensemble and indicate with Ψ^{corr} the indicator Ψ calculated on the corresponding uncorrelated multiplex ensemble. Finally, to quantify the additional amount of information carried by the correlated multiplex ensemble with respect to the uncorrelated multiplex ensemble, we measure the indicator Ξ as

$$\Xi = \frac{\Psi^{corr}}{\Psi^{uncorr}}. \tag{50}$$

The finite-size scaling of Ψ^{corr}, Ψ^{corr} and Ξ are shown in Fig. 4 in the manuscript.

[1] M. E. J. Newman (2001) *Phys. Rev. E* 64:016132.
[2] A.Barrat, M. Barthélemy, R. Pastor-Satorras,A. Vespignani (2004) *PNAS* 101: 3747.
[3] http://www.mathworks.co.uk/help/stats/regress.html
[4] A. Barrat, M. Barthélemy, A. Vespignani (2008) *Dynamical Processes on complex Networks* (Cambridge University Press, Cambridge).
[5] J. Park and M. E. J. Newman (2004) *Phys. Rev. E* 70:066117.
[6] G. Bianconi (2013) *Phys. Rev. E* 87:062806.

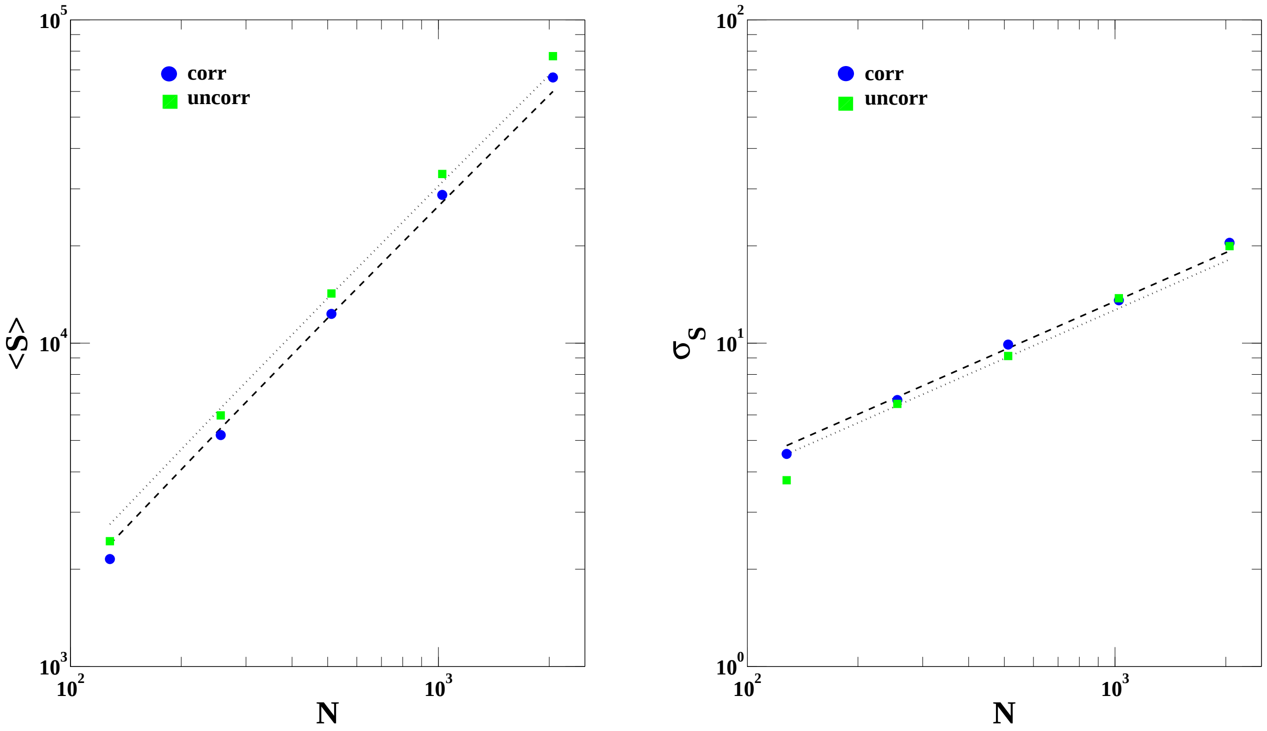

FIG. 6: The mean $\langle \mathcal{S} \rangle$ and variance σ_S as a function of the system size N for the null models of correlated and uncorrelated multiplex ensembles in which the weights are distributed uniformly over the links of the multiplex network. The solid lines indicate the fit of the data in which we assume $\langle \mathcal{S} \rangle = aN \log N$ and $\sigma_S = b\sqrt{N}$.
